# A new flexible odd type-G family of distributions with properties and applications in the biomedical sector

**DOI:** 10.1038/s41598-025-19351-6

**Published:** 2025-10-10

**Authors:** Zubir Shah, Dost Muhammad Khan, Imad Khan, Saddam Hussain, Fatimah M. Alghamdi, Hassan M. Aljohani

**Affiliations:** 1 Department of Statistics, Abdul Wali Khan University, Mardan, 23200 Pakistan; 2 ARIA University Balkh, Mazar-i-Sharif, Afghanistan; 3 Department of Mathematical Sciences, Princess Nourah Bint Abdulrahman University, P.O. Box 84428, Riyadh, 11671 Saudi Arabia; 4 Department of Mathematics and Statistics, College of Science, Taif University, P.O. Box 11099, Taif, 21944 Saudi Arabia

**Keywords:** Biomedical data analysis, Estimation, Monte carlo simulation analysis, New flexible odd type-G family, Statistical properties, Weibull distribution, Health care, Applied mathematics, Scientific data, Statistics

## Abstract

In this paper, we propose a new family of probability distributions called the new flexible odd type-G family of distributions. We have used the Weibull distribution as the base reference to introduce a new heavy-tailed distribution, which we have fittingly named the new flexible odd-type Weibull distribution. The hazard function of the newly proposed distribution exhibits various shapes, such as increasing, decreasing, unimodal, S-shaped, J-shaped, reverse J-shaped, and bathtub shapes. For the proposed distribution, we have derived various distributional properties, including moments, moment generating function, characteristic function, mean deviation from median, and order statistics. Furthermore, actuarial measures, such as Value at Risk and Tail Value at Risk, are calculated, and it is empirically demonstrated that the proposed distribution exhibits heavy-tailed behavior. To estimate the model parameter, we applied the maximum likelihood estimation method and conducted a simulation study to assess its performance for the proposed distribution. From the simulation results, it is confirmed that as the sample size increases, the mean square error and biases decrease and approach zero. Finally, we evaluated three real data sets from the biomedical field to demonstrate the flexibility and superiority of the proposed distribution in comparison to the other nine probability distributions. The analyzed results (numerically as well as graphically) show that the newly proposed model provides a superior fit compared to the competing distributions.

## Introduction

Probability distributions are a key component of statistics, playing a crucial role in describing and modeling real-world data scenarios. Specifically, it is essential to identify the appropriate probability distributions to gain a deeper understanding and effectively model real-world problems. Selecting the right probability distribution results in an accurate and dependable estimate. Numerous probability distributions have been developed to address data scenarios across various fields, including business, economics, physical and biological sciences, education, behavioral sciences, engineering, and information technology. The diversity of applied fields generates a broad array of data scenarios, prompting statisticians to explore and create new probability distributions to more effectively model these situations.

The availability of positively skewed data scenarios is most prevalent in applied research fields. The Weibull, Rayleigh, gamma, and exponential distributions are the most commonly used probability distributions for modeling such situations. Unfortunately, the reliability analysis and positively skewed scenarios are beyond the scope of these models. For instance, in the distribution theory literature, the exponential distribution is the most widely used; however, it cannot effectively model data with a non-constant hazard function (HF). The exponential distribution can only model data scenarios with a constant HF; on the other hand, the Rayleigh distribution is limited to modeling data with an increasing HF. The gamma distribution can represent increasing and decreasing HF and has wide applications besides survival analysis. However, a major drawback of the gamma distribution is that its distribution and survival functions cannot be stated in closed form, requiring numerical integration to determine their mathematical properties. Similarly, the Weibull model is the most commonly used distribution in the literature, incorporating characteristics of both the gamma and exponential models. The Weibull model cannot handle data with non-monotonic (unimodal, bathtub-shaped, and modified unimodal) HF, as it is only capable of modeling situations with monotonic (increasing, decreasing, or constant) HF; see references Almalki and Yuan^1^ and Liao et al.^2^ for further details. To address these challenges, researchers have been continually working to propose new distributions by building on existing distributions as their baseline. The Weibull distribution is the ideal choice as a baseline distribution for proposing new flexible distributions. Looking back at the literature review, we can see that various families (or methods) have been proposed for deriving new versatile distributions using the Weibull distribution as a base distribution. For instance, Mudholkar and Srivastava^3^ proposed an Exponentiated family and considered a modified form of Weibull distribution, Marshall and Olkin^4^ proposed a Marshall Olkin family of distributions and used the Weibull model as a base distribution, Mahdavi and Kundu^5^ suggested alpha-power-transformation family of distributions and based on this method Dey et al.^6^ derived a modified form of the Weibull distribution, Bantan et al.^7^ developed the truncated Burr XG distribution family for analyzing actuarial and financial data, Reyad et al.^8^ proposed the Fréchet Topp Leone-G family of probability distributions, Huo et al.^9^ proposed a new lifetime exponential-X family of distribution for analyzing reliability data, Alkhairy et al.^10^ developed a new flexible logarithmic-X method of distributions, Shah et al.^11^ suggested a new MEA (modified exponent alpha) family of distributions for analyzing reliability engineering data, Shah et al.^12^ derived a novel flexible exponent power-X family of distributions with applications to COVID-19 mortality rate in Mexico and Canada, Eghwerido et al.^13^ introduced the shifted Gompertz-G family of distributions, Tung et al.^14^ suggested the Arcsine-X family of distributions, and Shah et al.^15^ proposed a new flexible exponent power family of distributions with biomedical data analysis. For further details about the proposed families of distributions, please see the references, Gillariose et al.^16^, Gillariose et al.^17^, El-Alosey et al., (2024)^18^, Qayoom et al.^19^, Alosey et al.^20^, Alharbi et al.^21^, Ahmad et al.^22^, Gemeay et al.^23^, Yıldırım et al.^24^, Teamah et al.^25^, Gemeay et al.^26^, Shah et al.^27^, and Hassan et al.^28^.

Alnssyan et al.^29^ developed a new family, namely, the novel updated-W (NU-W) method of probability distributions. The NU-W family is used for updating the Weibull distribution, and its CDF (cumulative distribution function) can be expressed as

1 $$F\left( {w;\delta ,\alpha ,\phi } \right)=1 - \left[ {\frac{{1 - G\left( {w;\phi } \right)}}{\delta }\left\{ {\delta - {{\left( {G\left( {w;\phi } \right)} \right)}^\alpha }} \right\}} \right];\quad w \in \Re ,$$

where, $\delta >0$, and $\alpha >0$are the additional parameters. $G\left( {w;\phi } \right)$is the CDF of any base distribution with the attached parameter vector $\phi$. Recently, Zhao et al.^30^ proposed a novel logarithmic-U family of probability distributions, using the Weibull distribution as a base distribution to obtain its modified version. The CDF of their proposed method can be expressed as

2 $$F\left( {w;\alpha ,\sigma ,\phi } \right)=1 - {\left( {1 - \frac{{\alpha G\left( {w;\phi } \right)}}{{\alpha - \log G\left( {w;\phi } \right)}}} \right)^\sigma };\quad w \in \Re ,$$

where, $\sigma >0$ and $\alpha >0$are the additional parameters. $G\left( {w;\phi } \right)$is the CDF of any base distribution with the attached parameter vector $\phi$.

Similarly, several modifications of the Weibull distribution have been introduced in the literature. However, these modifications are not able to capture up-to-the-minute the true characteristics inherent in their stochastic scenarios. To address these challenges, in this article, we present a new, practical, and improved version of the Weibull distribution to bridge the gaps of constant failure and survival rates that do exist. This improved model is derived by using a novel method known as the New Flexible Odd-Type-G (NFOT-G) family of distributions. The newly proposed distribution is called the New Flexible Odd-Type Weibull (NFOT-W) distribution. This introduced distribution is more flexible and offers a variety of failure rate functions, including increasing, decreasing, J-shaped, inverted J-shaped, S-shaped, and bathtub-shaped patterns. Thus, the newly introduced family provides a foundation for developing other probability distributions for modeling complex data patterns in the future.

The subsequent sections of this article are organized as follows: “NFOT-G family of distributions (NFOT-GFD)” introduces the NFOT-G family of probability distributions. In “NFOT-W distribution (NFOT-WD)”, we discuss the NFOT-W distribution and the graphical representations of its PDF (probability density function) and the HF. “Statistical properties of the NFOT-WD” derives some basic distributional properties of the NFOT-W distribution. The actuarial measures, such as VaR and TVaR, are evaluated in “Actuarial measures”. The estimation of the unknown parameters for the NFOT-W distribution is presented in “Maximum likelihood method”. “Simulations” provides a simulation analysis to assess the practical performance of the applied estimation method. “Applications” focuses on the applications of the NFOT-W distribution by analyzing three biomedical data sets. Finally, in “Conclusion”, some concluding remarks about the main work are offered.

## NFOT-G family of distributions (NFOT-GFD)

Here, in the present section of the article, we define the new proposed family of distributions. Let $W\sim NFOT - GFD$with parameter $\delta$, then its CDF and PDF of the NFOT-GFD, signified by $F\left( {w;\phi } \right)$ and $f\left( {w;\phi } \right)$ respectively, are presented by

3 $$F\left( {w;\delta ,\phi } \right)={\left( {\pi - 1} \right)^\delta }{\left( {\frac{{G\left( {w;\phi } \right)}}{{\pi - G\left( {w;\phi } \right)}}} \right)^\delta };\quad w \in \Re ,$$

4 $$f\left( {w;\delta ,\phi } \right)=\frac{{\pi \delta {{\left( {\pi - 1} \right)}^\delta }g\left( {w;\phi } \right)G{{\left( {w;\phi } \right)}^{\delta - 1}}}}{{{{\left( {\pi - G\left( {w;\phi } \right)} \right)}^{\delta +1}}}};\quad w \in \Re ,$$

where, $G\left( {w;\phi } \right)$ and $g\left( {w;\phi } \right)$ represents the CDF and PDF of the base distribution with the corresponding parameter vector $\phi \in {\Re ^+}$. Similarly, linked to Eq. (3) and Eq. (4), the SF (survival function) is signified as$S\left( {w;\delta ,\phi } \right)=1 - F\left( {w;\delta ,\phi } \right)$, and the HF signified as $h\left( {w;\delta ,\phi } \right)=\frac{{f\left( {w;\delta ,\phi } \right)}}{{S\left( {w;\delta ,\phi } \right)}}$ of the NFOT-GFD can be presented as follows

5 $$S\left( {w;\delta ,\phi } \right)=1 - {\left( {\pi - 1} \right)^\delta }{\left( {\frac{{G\left( {w;\phi } \right)}}{{\pi - G\left( {w;\phi } \right)}}} \right)^\delta };\quad w \in \Re ,$$

6 $$h\left( {w;\delta ,\phi } \right)=\frac{{\pi \delta {{\left( {\pi - 1} \right)}^\delta }g\left( {w;\phi } \right)G{{\left( {w;\phi } \right)}^{\delta - 1}}{{\left( {\pi - G\left( {w;\phi } \right)} \right)}^{ - 1}}}}{{{{\left( {\pi - G\left( {w;\phi } \right)} \right)}^\delta } - {{\left( {\pi - 1} \right)}^\delta }G{{\left( {w;\phi } \right)}^\delta }}};\quad w \in \Re ,$$

The QF (quantile function) and random deviation of the NFOT-GFD can be presented as follows

7 $${Q_w}\left( u \right)={G^{ - 1}}\left( {\frac{{\pi D}}{{1+D}}} \right);\;{\text{where}}\;D={\left\{ {u{{\left( {\pi - 1} \right)}^{ - \delta }}} \right\}^{\frac{1}{\delta }}}\;{\text{and}}\;u \in \left( {0,1} \right),$$

8 $$y={G^{ - 1}}\left( {\frac{{\pi A}}{{1+A}}} \right);\;{\text{where}}\;A={\left\{ {y{{\left( {\pi - 1} \right)}^{ - \delta }}} \right\}^{\frac{1}{\delta }}}.$$

Likewise, using some binomial expansion, the CDF in Eq. (3), of the NFOT-GFD can be expressed in linear form as

9 $$F\left( {w;\delta ,\phi } \right)=\sum\limits_{{k=0}}^{\infty } {{{\left( {\pi - 1} \right)}^\delta }{{\left( { - 1} \right)}^{\delta +k}}\left( {\begin{array}{*{20}{c}} \delta \\ k \end{array}} \right){{\left( {\frac{{G\left( {w;\phi } \right)}}{\pi }} \right)}^k}} .$$

The PDF of the NFOT-GFD corresponding to Eq. (9) can be written in linear form as

10 $$\begin{gathered} f\left( {w;\delta ,\phi } \right)=\sum\limits_{{k=0}}^{\infty } {{{\left( { - 1} \right)}^{\delta +k}}k{\pi ^{ - k}}{{\left( {\pi - 1} \right)}^\delta }\left( {\begin{array}{*{20}{c}} \delta \\ k \end{array}} \right){{\left( {G\left( {w;\phi } \right)} \right)}^{k - 1}}g\left( {w;\phi } \right)} ; \hfill \\ f\left( {w;\delta ,\phi } \right)=\sum\limits_{{k=0}}^{\infty } {{\Phi _k}{{\left( {G\left( {w;\phi } \right)} \right)}^{k - 1}}g\left( {w;\phi } \right)} ; \hfill \\ \end{gathered}$$

where, $${\Phi _k}={\left( { - 1} \right)^{\delta +k}}{\left( {\pi - 1} \right)^\delta }k{\pi ^{ - k}}\left( {\begin{array}{*{20}{c}} \delta \\ k \end{array}} \right)$$ and $$g\left( {w;\phi } \right)=\frac{d}{{dw}}G\left( {w;\phi } \right)$$.

## NFOT-W distribution (NFOT-WD)

In this section, we suggest a new flexible heavy-tailed distribution by utilizing the Weibull distribution as a base distribution. The corresponding CDF and PDF of the Weibull distribution with parameters$\omega >0$ and $\rho >0$ for $w>0$ are stated as $G\left( {w;\phi } \right)=1 - {e^{ - \omega {w^\rho }}}$ and $g\left( {w;\phi } \right)=\omega \rho {w^{\rho - 1}}{e^{ - \omega {w^\rho }}}$. Substituting the CDF $G\left( {w;\phi } \right)$and PDF $g\left( {w;\phi } \right)$ in Eqs. (3) and (4), then we obtain the CDF$F\left( {w;\delta ,\phi } \right)$ and PDF $f\left( {w;\delta ,\phi } \right)$ of the NFOT-WD as follows

11 $$F\left( {w;\phi ,\delta } \right)={\left( {\pi - 1} \right)^\delta }{\left( {\frac{{1 - {e^{ - \omega {w^\rho }}}}}{{\pi - 1+{e^{ - \omega {w^\rho }}}}}} \right)^\delta };\quad \delta ,\omega ,\rho \in {\Re ^+},\;w \in {\Re ^+},$$

12 $$f\left( {w;\delta ,\phi } \right)=\frac{{\delta \omega \rho \pi {{\left( {\pi - 1} \right)}^\delta }{w^{\rho - 1}}{e^{ - \omega {w^\rho }}}{{\left( {1 - {e^{ - \omega {w^\rho }}}} \right)}^{\delta - 1}}}}{{{{\left( {\pi - 1+{e^{ - \omega {w^\rho }}}} \right)}^{\delta +1}}}};\quad w \in {\Re ^+}.$$

Furthermore, the SF $S\left( {w;\delta ,\phi } \right)$ and HF $h\left( {w;\delta ,\phi } \right)$, of the NFOT-WD are expressed as follows

13 $$S\left( {w;\delta ,\phi } \right)=1 - {\left( {\pi - 1} \right)^\delta }{\left( {\frac{{1 - {e^{ - \omega {w^\rho }}}}}{{\pi - 1+{e^{ - \omega {w^\rho }}}}}} \right)^\delta };\quad w \in {\Re ^+},$$

14 $$h\left( {w;\delta ,\phi } \right)=\frac{{\pi \delta \omega \rho {{\left( {\pi - 1} \right)}^\delta }{w^{\rho - 1}}{e^{ - \omega {w^\rho }}}{{\left( {1 - {e^{ - \omega {w^\rho }}}} \right)}^{\delta - 1}}{{\left( {\pi - 1+{e^{ - \omega {w^\rho }}}} \right)}^{ - 1}}}}{{{{\left( {\pi - 1+{e^{ - \omega {w^\rho }}}} \right)}^\delta } - {{\left( {\pi - 1} \right)}^\delta }{{\left( {1 - {e^{ - \omega {w^\rho }}}} \right)}^\delta }}};\quad w \in {\Re ^+}.$$

**Fig. 1:**
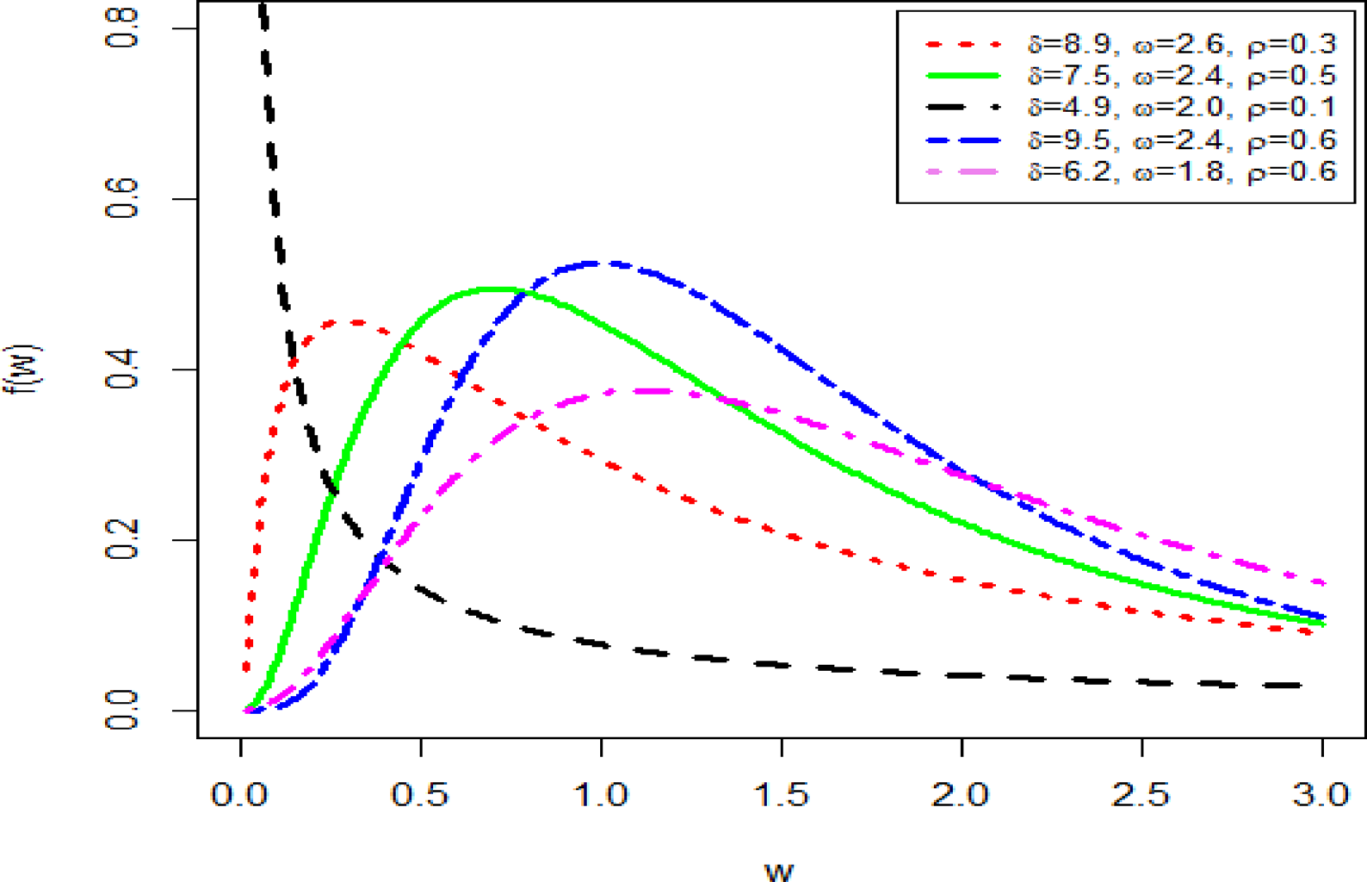
Different shapes of the PDF $f\left( {w;\delta ,\phi } \right)$ of the NFOT-WD distribution for distinct choices of the parameter values.

**Fig. 2:**
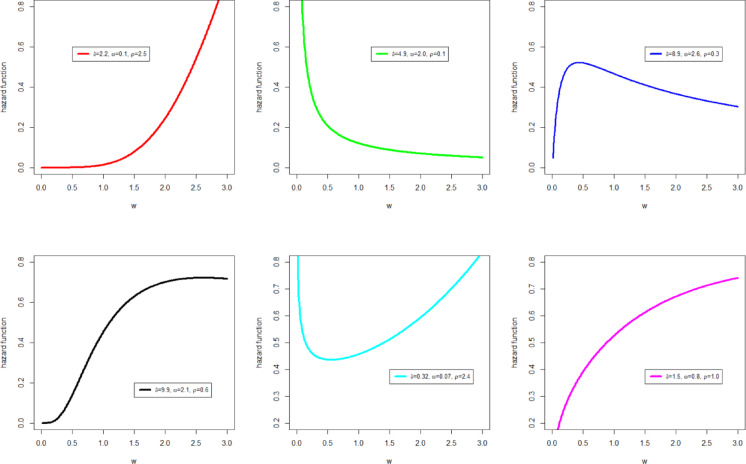
Different shapes of the HF$h\left( {w;\delta ,\phi } \right)$ of the NFOT-WD distribution for distinct choices of the parameter values.

The PDF and HF shapes of the NFOT-WD distribution are graphically illustrated in Figs. 1 and 2, respectively. In Fig. 1, the PDF$f\left( {w;\delta ,\phi } \right)$ of the NFOT-WD distribution can exhibit different behaviors, such as positively skewed and reversed J-shaped. Similarly, in Fig. 2, the HF $h\left( {w;\delta ,\phi } \right)$ of the NFOT-WD distribution can display a range of non-monotonic shapes, including increasing, decreasing, reversed J-shaped, J-shaped, S-shaped, and bathtub shapes. These visual illustrations of the NFOT-WD can be observed as clear measures of a high level of adaptability of these functions.

Furthermore, let U be a random variable that follows the NFOT-WD distribution with $u \in \left( {0,1} \right)$, then the QF, say ${Q_w}\left( u \right)$can be expressed in the following form

15 $${Q_w}\left( u \right)={\left\{ {\frac{{ - 1}}{\omega }\log \left( {\frac{{1 - \left( {\pi - 1} \right)H}}{{1+H}}} \right)} \right\}^{\frac{1}{\rho }}};\quad H={\left\{ {u{{\left( {\pi - 1} \right)}^{ - \delta }}} \right\}^{\frac{1}{\delta }}}.$$

The expression in Eq. (15) represents the inverse of the CDF in Eq. (11), which is used to generate random samples from the NFOT-WD.

## Statistical properties of the NFOT-WD

### Linear form of the CDF and PDF of the NFOT-WD

Using algebraic expansions, the PDF of the NFOT-WD derived in Eq. (12) can be expressed in linear form as

16 $$\begin{gathered} f\left( {w;\delta ,\phi } \right)=\sum\limits_{{k=0}}^{\infty } {\omega \rho {\Phi _k}{{\left( {1 - {e^{ - \omega {w^\rho }}}} \right)}^{k - 1}}{w^{\rho - 1}}{e^{ - \omega {w^\rho }}}} ; \hfill \\ f\left( {w;\delta ,\phi } \right)=\sum\limits_{{k=0}}^{\infty } {\sum\limits_{{j=0}}^{\infty } {\Phi _{k}^{*}{w^{\rho - 1}}{e^{ - \left( {j+1} \right)\omega {w^\rho }}}} } ; \hfill \\ \end{gathered}$$

where, $$\Phi _{k}^{*}={\left( { - 1} \right)^j}\omega \rho {\Phi _k}\left( {\begin{array}{*{20}{c}} {k - 1} \\ j \end{array}} \right)$$ and $${\Phi _k}={\left( { - 1} \right)^{\delta +k}}k{\pi ^{ - k}}{\left( {\pi - 1} \right)^\delta }\left( {\begin{array}{*{20}{c}} \delta \\ k \end{array}} \right)$$.

### Moments

Let W be a random variable that follows$NFOTWD\left( {\delta ,\omega ,\rho } \right)$, then the r^th^ moments corresponding to Eq. (16) can be obtained as

17 $$\begin{gathered} E\left( {{W^r}} \right)=\sum\limits_{{k=0}}^{\infty } {\sum\limits_{{j=0}}^{\infty } {\Phi _{k}^{*}\int\limits_{0}^{\infty } {{w^{\rho +r - 1}}{e^{ - \left( {j+1} \right)\omega {w^\rho }}}dw} } } ; \hfill \\ E\left( {{W^r}} \right)=\sum\limits_{{k=0}}^{\infty } {\sum\limits_{{j=0}}^{\infty } {\Phi _{k}^{*}\int\limits_{0}^{\infty } {{x^{\frac{r}{\rho }+1 - 1}}{e^{ - \left( {j+1} \right)\omega x}}dx} } } ; \hfill \\ E\left( {{W^r}} \right)=\sum\limits_{{k=0}}^{\infty } {\sum\limits_{{j=0}}^{\infty } {\Phi _{k}^{*}\frac{{{\omega ^{ - 1}}\Gamma \left( {\frac{r}{\rho }+1} \right)}}{{{{\left\{ {\left( {j+1} \right)\omega } \right\}}^{\frac{r}{\rho }+1}}}}} } . \hfill \\ \end{gathered}$$

The mean, the first four raw moments, and the variance of the NFOT-WD, using Eq. (17), can be derived from the following expressions, and the numerical results to illustrate the mean, second raw moment, variance, skewness, and kurtosis are displayed in Table 1.

18 $$E\left( {{W^1}} \right)={\mu ^{\prime}_1}=\sum\limits_{{k=0}}^{\infty } {\sum\limits_{{j=0}}^{\infty } {\Phi _{k}^{*}\frac{{{\omega ^{ - 1}}\Gamma \left( {\frac{{1+\rho }}{\rho }} \right)}}{{{{\left\{ {\left( {j+1} \right)\omega } \right\}}^{\frac{{1+\rho }}{\rho }}}}}} } ;$$

19 $$E\left( {{W^2}} \right)={\mu ^{\prime}_2}=\sum\limits_{{k=0}}^{\infty } {\sum\limits_{{j=0}}^{\infty } {\Phi _{k}^{*}\frac{{{\omega ^{ - 1}}\Gamma \left( {\frac{{2+\rho }}{\rho }} \right)}}{{{{\left\{ {\left( {j+1} \right)\omega } \right\}}^{\frac{{2+\rho }}{\rho }}}}}} } ;$$

20 $$E\left( {{W^3}} \right)={\mu ^{\prime}_3}=\sum\limits_{{k=0}}^{\infty } {\sum\limits_{{j=0}}^{\infty } {\Phi _{k}^{*}\frac{{{\omega ^{ - 1}}\Gamma \left( {\frac{{3+\rho }}{\rho }} \right)}}{{{{\left\{ {\left( {j+1} \right)\omega } \right\}}^{\frac{{3+\rho }}{\rho }}}}}} } ;$$

and

21 $$E\left( {{W^4}} \right)={\mu ^{\prime}_4}=\sum\limits_{{k=0}}^{\infty } {\sum\limits_{{j=0}}^{\infty } {\Phi _{k}^{*}\frac{{{\omega ^{ - 1}}\Gamma \left( {\frac{{4+\rho }}{\rho }} \right)}}{{{{\left\{ {\left( {j+1} \right)\omega } \right\}}^{\frac{{4+\rho }}{\rho }}}}}} } .$$

The variance can be computed as

22 $$V\left( W \right)=\sum\limits_{{k=0}}^{\infty } {\sum\limits_{{j=0}}^{\infty } {\Phi _{k}^{*}\frac{{{\omega ^{ - 1}}\Gamma \left( {\frac{{2+\rho }}{\rho }} \right)}}{{{{\left\{ {\left( {j+1} \right)\omega } \right\}}^{\frac{{2+\rho }}{\rho }}}}}} } - {\left[ {\sum\limits_{{k=0}}^{\infty } {\sum\limits_{{j=0}}^{\infty } {\Phi _{k}^{*}\frac{{{\omega ^{ - 1}}\Gamma \left( {\frac{{1+\rho }}{\rho }} \right)}}{{{{\left\{ {\left( {j+1} \right)\omega } \right\}}^{\frac{{1+\rho }}{\rho }}}}}} } } \right]^2}.$$

**Table 1:** Some descriptive measures with different combinations of parameter values.

$\delta$	$\omega$	$\rho$	$E\left( w \right)$	$E\left( {{w^2}} \right)$	$\operatorname{var} \left( w \right)$	Skewness	Kurtosis
0.300	0.500	0.500	3.662446	169.8959	156.4824	9.466387	173.9393
0.300	0.500	0.750	1.440720	11.43004	9.354363	4.420600	33.75945
0.300	0.500	1.500	0.786746	1.440719	0.821750	1.659169	6.079872
0.300	0.750	0.500	1.627754	33.55968	30.91010	9.466409	173.9392
0.300	0.750	0.750	0.839056	3.876778	3.172762	4.420599	33.75945
0.300	0.750	1.500	0.600399	0.839056	0.478576	1.659169	6.079872
0.300	1.500	0.500	0.406938	2.097486	1.931881	9.466413	173.9392
0.300	1.500	0.750	0.332979	0.610554	0.499678	4.420632	33.75945
0.300	1.500	1.500	0.378228	0.332979	0.189923	1.659169	6.079873
0.500	0.500	0.500	5.837739	280.4275	246.3483	7.562028	112.6054
0.500	0.500	0.750	2.216285	18.55752	13.64564	3.547411	23.11704
0.500	0.500	1.500	1.114541	2.216285	0.974083	1.244346	4.659558
0.500	0.750	0.500	2.594551	55.39309	48.66145	7.562046	112.6054
0.500	0.750	0.750	1.290735	6.294243	4.628242	3.547411	23.11704
0.500	0.750	1.500	0.850554	1.290735	0.567294	1.244345	4.659557
0.500	1.500	0.500	0.648638	3.462068	3.041337	7.562052	112.6054
0.500	1.500	0.750	0.512228	0.991281	0.728903	3.547411	23.11704
0.500	1.500	1.500	0.535815	0.512228	0.225130	1.244346	4.659558
0.900	0.500	0.500	9.707043	495.5592	401.3325	5.954684	71.47437
0.900	0.500	0.750	3.482323	31.83929	19.71272	2.826613	16.10755
0.900	0.500	1.500	1.558962	3.482322	1.051961	0.925255	3.946709
0.900	0.750	0.500	4.314241	97.88823	79.27556	5.954700	71.47433
0.900	0.750	0.750	2.028059	10.79908	6.686055	2.826612	16.10755
0.900	0.750	1.500	1.189710	2.028059	0.612649	0.925256	3.946709
0.900	1.500	0.500	1.078560	6.118014	4.954722	5.954703	71.47433
0.900	1.500	0.750	0.804835	1.700748	1.052988	2.826613	16.10755
0.900	1.500	1.500	0.749471	0.804835	0.243129	0.925255	3.946709

Table 1 reveals that the mean, second raw moment, and variance of the NFOT-WD decrease for fixed $\delta$ and $\omega$ with increases in $\rho$. However, for fixed $\delta$ and $\omega$ with increases in $\rho$, the skewness and kurtosis values of the NFOT-WD decrease. Similarly, at a fixed value of $\omega$ and $\rho$, the skewness and kurtosis of the NFOT-WD also decrease with increasing $\delta$. Furthermore, as shown in Table 1, the NFOT-WD exhibits positive skewness and a leptokurtic distribution, as all the skewness values exceed zero (skewness > 0) and all the kurtosis values are greater than three (kurtosis > 3). Hence, these results indicate that the proposed distribution is more flexible and can be used as a good candidate for modeling positively skewed data sets.

### Moments generating (MG) function

MG function can be used to calculate various moments and their mathematical expression, say ${M_w}(t)$can be formulated by using Eq. (16) as follows

23 $$\begin{gathered} {M_w}\left( t \right)=\sum\limits_{{k=0}}^{\infty } {\sum\limits_{{j=0}}^{\infty } {\sum\limits_{{r=0}}^{\infty } {\Phi _{k}^{*}\frac{{{t^r}}}{{r!}}\int\limits_{0}^{\infty } {{w^{\rho +r - 1}}{e^{ - \left( {j+1} \right)\omega {w^\rho }}}dw} } } } ; \hfill \\ {M_w}\left( t \right)=\sum\limits_{{k=0}}^{\infty } {\sum\limits_{{j=0}}^{\infty } {\sum\limits_{{r=0}}^{\infty } {\Phi _{k}^{*}\frac{{{t^r}}}{{r!}}\int\limits_{0}^{\infty } {{x^{\frac{r}{\rho }+1 - 1}}{e^{ - \left( {j+1} \right)\omega x}}dx} } } } ; \hfill \\ {M_w}\left( t \right)=\sum\limits_{{k=0}}^{\infty } {\sum\limits_{{j=0}}^{\infty } {\sum\limits_{{r=0}}^{\infty } {\Phi _{k}^{*}\frac{{{t^r}}}{{r!}}\frac{{{\rho ^{ - 1}}\Gamma \left( {\frac{r}{\rho }+1} \right)}}{{{{\left\{ {\left( {j+1} \right)\omega } \right\}}^{\frac{r}{\rho }+1}}}}} } } . \hfill \\ \end{gathered}$$

### Characteristics function (CF)

The CF allows us to explore the properties of distributions and their mathematical expression, say ${\Upsilon _w}\left( t \right)$can be obtained by using Eq. (16), as follows

24 $$\begin{gathered} {\Upsilon _w}\left( t \right)=\sum\limits_{{k=0}}^{\infty } {\sum\limits_{{j=0}}^{\infty } {\sum\limits_{{k=0}}^{\infty } {\Phi _{k}^{*}\frac{{{{\left( {zt} \right)}^k}}}{{k!}}\int\limits_{0}^{\infty } {{w^{\rho +k - 1}}{e^{ - \left( {j+1} \right)\omega {w^\rho }}}dw} } } } ; \hfill \\ {\Upsilon _w}\left( t \right)=\sum\limits_{{k=0}}^{\infty } {\sum\limits_{{j=0}}^{\infty } {\sum\limits_{{k=0}}^{\infty } {\Phi _{k}^{*}\frac{{{{\left( {zt} \right)}^k}}}{{k!}}\int\limits_{0}^{\infty } {{x^{\frac{k}{\rho }+1 - 1}}{e^{ - \left( {j+1} \right)\omega x}}dx} } } } ; \hfill \\ {\Upsilon _w}\left( t \right)=\sum\limits_{{k=0}}^{\infty } {\sum\limits_{{j=0}}^{\infty } {\sum\limits_{{k=0}}^{\infty } {\Phi _{k}^{*}\frac{{{{\left( {zt} \right)}^k}}}{{k!}}\frac{{{\rho ^{ - 1}}\Gamma \left( {\frac{k}{\rho }+1} \right)}}{{{{\left\{ {\left( {j+1} \right)\omega } \right\}}^{\frac{k}{\rho }+1}}}}} } } ; \hfill \\ \end{gathered}$$

where, $$z=\sqrt { - 1}$$.

### Mean dDeviation (MD) from the median

The MD, say$M.D\left( W \right)$from the median (Med) of the NFOT-WD can be calculated as

25 $$\begin{gathered} M.D\left( W \right)=\int\limits_{0}^{{Med}} {\left( {Med - w} \right)} f\left( {w;\delta ,\phi } \right)dw+\int\limits_{{Med}}^{\infty } {\left( {w - Med} \right)} f\left( {w;\delta ,\phi } \right)dw; \hfill \\ M.D\left( W \right)=MedF\left( {Med} \right) - Med - \mu +2\int\limits_{{Med}}^{\infty } w f\left( {w;\delta ,\phi } \right)dw; \hfill \\ M.D\left( W \right)=MedF\left( {Med} \right) - Med - \mu +2\sum\limits_{{k=0}}^{\infty } {\sum\limits_{{j=0}}^{\infty } {\Phi _{k}^{*}\frac{{{\rho ^{ - 1}}\Gamma \left( {\frac{{1+\rho }}{\rho },\left( {j+1} \right)\omega Med} \right)}}{{{{\left\{ {\left( {j+1} \right)\omega } \right\}}^{\frac{{1+\rho }}{\rho }}}}}} } ; \hfill \\ \end{gathered}$$

where $\mu$is the mean value of the random variable w, $\Gamma \left\{ {.,.} \right\}$is the upper incomplete gamma function, and $F\left( . \right)$is the CDF of the NFOT-WD presented in Eq. (11).

### Order statistics

Let ${W_1},{W_2},\ldots ,{W_n}$ be the random samples of size n with observed sample ${w_1},{w_2},\ldots ,{w_n}$drawn from an NFOT-WD with CDF $F\left( {w;\delta ,\phi } \right)$ and PDF $f\left( {w;\delta ,\phi } \right)$. If${f_{k:n}}\left( w \right)$ denotes the PDF of k^th^ order statistic ${W_{\left( k \right)}}$, then their PDF is given by

26 $${f_{k:n}}\left( v \right)=\frac{{n!}}{{\left( {k - 1} \right)!\left( {n - k} \right)!}}F{\left( {w;\delta ,\phi } \right)^{k - 1}}f\left( {w;\delta ,\phi } \right){\left( {1 - F\left( {w;\delta ,\phi } \right)} \right)^{n - k}}.$$

Now, by putting Eq. (11) and Eq. (12) in Eq. (26), we get

27 $$\begin{aligned} f_{{k:n}} \left( w \right) &= \frac{{n!}}{{\left( {k - 1} \right)!\left( {n - k} \right)!}}\frac{{\pi \delta \omega \rho \left( {\pi - 1} \right)^{\delta } w^{{\rho - 1}} e^{{ - \omega w^{\rho } }} \left( {1 - e^{{ - \omega w^{\rho } }} } \right)^{{\delta - 1}} }}{{\left( {\pi - 1 + e^{{ - \omega w^{\rho } }} } \right)^{{\delta + 1}} }} \hfill \\ &\quad \times \left[ {\left( {\pi - 1} \right)^{\delta } \left\{ {\frac{{\left( {1 - e^{{ - \omega w^{\rho } }} } \right)}}{{\pi - \left( {1 - e^{{ - \omega w^{\rho } }} } \right)}}} \right\}^{\delta } } \right]^{{k - 1}} \left[ {1 - \left( {\pi - 1} \right)^{\delta } \left\{ {\frac{{\left( {1 - e^{{ - \omega w^{\rho } }} } \right)}}{{\pi - \left( {1 - e^{{ - \omega w^{\rho } }} } \right)}}} \right\}^{\delta } } \right]^{{n - k}} . \hfill \\ \end{aligned}$$

The CDF ${F_{1:n}}\left( w \right)$ and PDF ${f_{1:n}}\left( w \right)$ of the first-order statistics ${W_{\left( 1 \right)}}$, can be expressed as

28 $${F_{1:n}}\left( w \right)=1 - {\left\{ {1 - {{\left( {\pi - 1} \right)}^\delta }{{\left( {\frac{{1 - {e^{ - \omega {w^\rho }}}}}{{\pi - 1+{e^{ - \omega {w^\rho }}}}}} \right)}^\delta }} \right\}^n};$$

and

29 $${f_{1:n}}\left( w \right)=\frac{{\pi n\delta \omega \rho {{\left( {\pi - 1} \right)}^\delta }{w^{\rho - 1}}{e^{ - \omega {w^\rho }}}{{\left( {1 - {e^{ - \omega {w^\rho }}}} \right)}^{\delta - 1}}}}{{{{\left( {\pi - 1+{e^{ - \omega {w^\rho }}}} \right)}^{\delta +1}}}}{\left[ {1 - {{\left( {\pi - 1} \right)}^\delta }{{\left\{ {\frac{{\left( {1 - {e^{ - \omega {w^\rho }}}} \right)}}{{\pi - \left( {1 - {e^{ - \omega {w^\rho }}}} \right)}}} \right\}}^\delta }} \right]^{n - 1}}.$$

The CDF${F_{n:n}}\left( w \right)$ and PDF ${f_{n:n}}\left( w \right)$ of the n^th^ order statistics ${W_{\left( n \right)}}$, can be expressed as

30 $${F_{n:n}}\left( w \right)={\left\{ {{{\left( {\pi - 1} \right)}^\delta }{{\left( {\frac{{1 - {e^{ - \omega {w^\rho }}}}}{{\pi - 1+{e^{ - \omega {w^\rho }}}}}} \right)}^\delta }} \right\}^n};$$

and

31 $${f_{n:n}}\left( w \right)=\frac{{\pi n\delta \omega \rho {{\left( {\pi - 1} \right)}^\delta }{w^{\rho - 1}}{e^{ - \omega {w^\rho }}}{{\left( {1 - {e^{ - \omega {w^\rho }}}} \right)}^{\delta - 1}}}}{{{{\left( {\pi - 1+{e^{ - \omega {w^\rho }}}} \right)}^{\delta +1}}}}{\left[ {{{\left( {\pi - 1} \right)}^\delta }{{\left\{ {\frac{{\left( {1 - {e^{ - \omega {w^\rho }}}} \right)}}{{\pi - \left( {1 - {e^{ - \omega {w^\rho }}}} \right)}}} \right\}}^\delta }} \right]^{n - 1}}.$$

## Actuarial measures

In this section, we present the mathematical and computational features of two well-known actuarial measures, the VaR (Value-at-Risk) and TVaR (Tail Value-at-Risk), for the NFOT-WD distribution.

### Value at risk

VaR is a standard measure for evaluating market risk, also referred to as the quantile risk measure or quantile premium principle. It represents the percentage of portfolio value loss that will equal or exceed only W percent of the time. It is generally specified with a confidence level q (typically 90%, 95%, or 99%). According to Artzner, (1999)^31^, the VaR is equal to the q^th^ quantile of the NFOT-WD and is given by

32 $$VaR\left( w \right)={\left[ {\frac{{ - 1}}{\omega }\log \left\{ {\frac{{1 - \left( {\pi - 1} \right){q^{\frac{1}{\delta }}}{{\left( {\pi - 1} \right)}^{ - 1}}}}{{1+{q^{\frac{1}{\delta }}}{{\left( {\pi - 1} \right)}^{ - 1}}}}} \right\}} \right]^{\frac{1}{\rho }}};\quad q \in \left( {0,1} \right).$$

### Tail value at risk

The TVaR is an essential risk measure and is effectively used for assessing the risk beyond a specific probability level that has taken place. The TVaR of a random variable W is defined as

33 $$TVaR\left( w \right)=\frac{1}{{1 - q}}\int\limits_{{VaR}}^{\infty } {wf\left( {w;\delta ,\phi } \right)dw} .$$

Substituting Eq. (16) in Eq. (33), we get

34 $$\begin{gathered} TVaR\left( w \right)=\frac{1}{{1 - q}}\sum\limits_{{k=0}}^{\infty } {\sum\limits_{{j=0}}^{\infty } {\Phi _{k}^{*}\int\limits_{{VaR}}^{\infty } {w{w^{\rho - 1}}{e^{ - \left( {j+1} \right)\omega {w^\rho }}}dw} } } ; \hfill \\ TVaR\left( w \right)=\frac{1}{{1 - q}}\sum\limits_{{k=0}}^{\infty } {\sum\limits_{{j=0}}^{\infty } {\Phi _{k}^{*}\int\limits_{{VaR}}^{\infty } {{w^{\frac{1}{\rho }+1 - 1}}{e^{ - \left( {j+1} \right)\omega x}}dx} } } ; \hfill \\ TVaR\left( w \right)=\frac{1}{{1 - q}}\sum\limits_{{k=0}}^{\infty } {\sum\limits_{{j=0}}^{\infty } {\Phi _{k}^{*}\frac{{{\rho ^{ - 1}}\Gamma \left( {\frac{{1+\rho }}{\rho },\left( {j+1} \right)\omega VaR} \right)}}{{{{\left\{ {\left( {j+1} \right)\omega } \right\}}^{\frac{{1+\rho }}{\rho }}}}}} } . \hfill \\ \end{gathered}$$

**Fig. 3:**
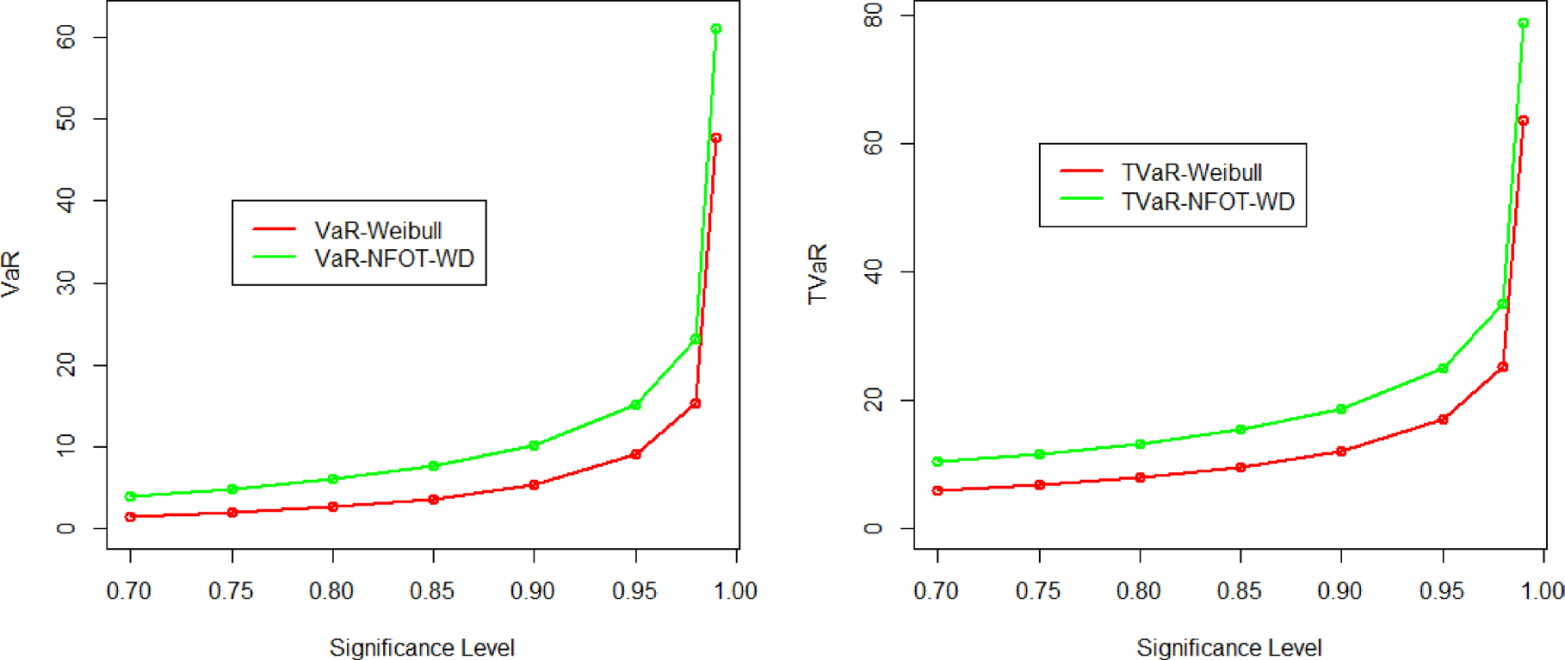
Pictorial illustration of the results recorded in Table 2.

The numerical values of the VaR and TVaR measures with different parameter values are presented in Table 2. Additionally, in support of these numerical values, the graphs of VaR and TVaR using the NFOT-WD and standard Weibull models are also illustrated in Fig. 3.

**Table 2:** Actuarial measures for $\delta =1.7$, $\omega =1.0$, and $\rho =0.5$.

Model	Parameters	Significance level	VaR	TVaR
Weibull	$\omega =1.0$ $\rho =0.5$	0.7000	1.44955	5.85750
		0.7500	1.92181	6.69440
		0.8000	2.59029	7.80917
		0.8500	3.59906	9.39331
		0.9000	5.30190	11.9071
		0.9500	8.97441	16.9659
		0.9800	15.3039	25.1280
		0.9999	47.7171	63.5325
NFOT-WD	$\delta =1.7$ $\omega =1.0$ $\rho =0.5$	0.7000	3.94226	10.3942
		0.7500	4.80546	11.6020
		0.8000	5.94651	13.1657
		0.8500	7.55459	15.3212
		0.9000	10.0839	18.6273
		0.9500	15.1271	25.0143
		0.9800	23.2105	34.8961
		0.9999	61.1678	78.8133

Generally, a model will be considered a heavy-tailed probability distribution if its risk assessment values are higher. The numerical values in Table 2 exhibit that the computed values of the NFOT-WD are higher than the standard Weibull distribution. Similarly, the graphical illustration in Fig. 3 also confirmed that the NFOT-WD is heavier-tailed than the standard Weibull distribution. Hence, the proposed distribution is more versatile and can be used as a good candidate model for modeling positively skewed data scenarios.

## Maximum likelihood method

To estimate the model parameter of the proposed distribution, the well-known method of estimation, i.e., MLE (Maximum Likelihood Estimation), is employed. Let ${W_1},{W_2},\ldots ,{W_n}$​ be the random samples of size n drawn from the NFOT-WD distribution, then the log-likelihood function linked to Eq. (12) is given by

35 $$L={\left( {\pi \delta \omega \rho } \right)^n}{\left( {\pi - 1} \right)^{n\delta }}\sum\limits_{{i=1}}^{n} {{w_i}^{{\rho - 1}}} {e^{ - \omega \sum\limits_{{i=1}}^{n} {{w_i}^{\rho }} }}\prod\limits_{{i=1}}^{n} {\frac{{{{\left( {1 - {e^{ - \omega {w_i}^{\rho }}}} \right)}^{\delta - 1}}}}{{{{\left( {\pi - 1+{e^{ - \omega {w_i}^{\rho }}}} \right)}^{\delta +1}}}}} .$$

Now, taking the logarithm of Eq. (35), we get

36 $$\log L=n\log \left( {\delta \omega \rho \pi } \right)+n\delta \log \left( {\pi - 1} \right)+\log \sum\limits_{{i=1}}^{n} {{w_i}^{{\rho - 1}}} - \omega \sum\limits_{{i=1}}^{n} {{w_i}^{\rho }} +\log \prod\limits_{{i=1}}^{n} {\frac{{{{\left( {1 - {e^{ - \omega {w_i}^{\rho }}}} \right)}^{\delta - 1}}}}{{{{\left( {\pi - 1+{e^{ - \omega {w_i}^{\rho }}}} \right)}^{\delta +1}}}}} .$$

Solving numerically $\frac{{\partial \log L\left( \Theta \right)}}{{\partial \delta }}$, $\frac{{\partial \log L\left( \Theta \right)}}{{\partial \omega }}$, and $\frac{{\partial \log L\left( \Theta \right)}}{{\partial \rho }}$simultaneously, we can get the MLEs of the parameter $\delta$, $\omega$, and $\rho$ symbolized by ${\hat {\delta }_{MLE}}$, ${\hat {\omega }_{MLE}}$, and ${\hat {\rho }_{MLE}}$, respectively.

## Simulations

Here, we use a simulation experiment to evaluate the performance of the applied maximum likelihood method for the NFOT-WD distribution. For this experiment, we have taken a set of random samples of size $n \in${25, 50, 100, 200, 300, 400, 500, 600, 700, 800, 900, 1000}, generated by using the inverse of the CDF of the NFOT-WD. The entire simulation process for each sample size is repeated 1,000 times. Four different sets of the combination of the initial values of parameters are chosen, such as Set-I ($\delta =2.5$, $\omega =3.5$, $\rho =2.5$), Set-II ($\delta =0.9$,$\omega =4.3$, $\rho =3.2$), Set-III ($\delta =1.6$,$\omega =1.7$,$\rho =4.7$), and Set-IV ($\delta =1.4$, $\omega =2.9$, $\rho =1.9$). We analyze the MLE estimates (onward, denoted by ${\Xi _{MLEs}}$), Biases (onward, denoted by ${\Xi _{Biases}}$), and (MSE) Mean Squared Error (onward, denoted by ${\Xi _{MSEs}}$) as key metrics to judge the performance of the applied estimation method for the NFOT-WD. The ${\Xi _{MLEs}}$, ${\Xi _{Biases}}$, and ${\Xi _{MSEs}}$are computed and numerically recorded in Tables 3 and 4.

**Fig. 4:**
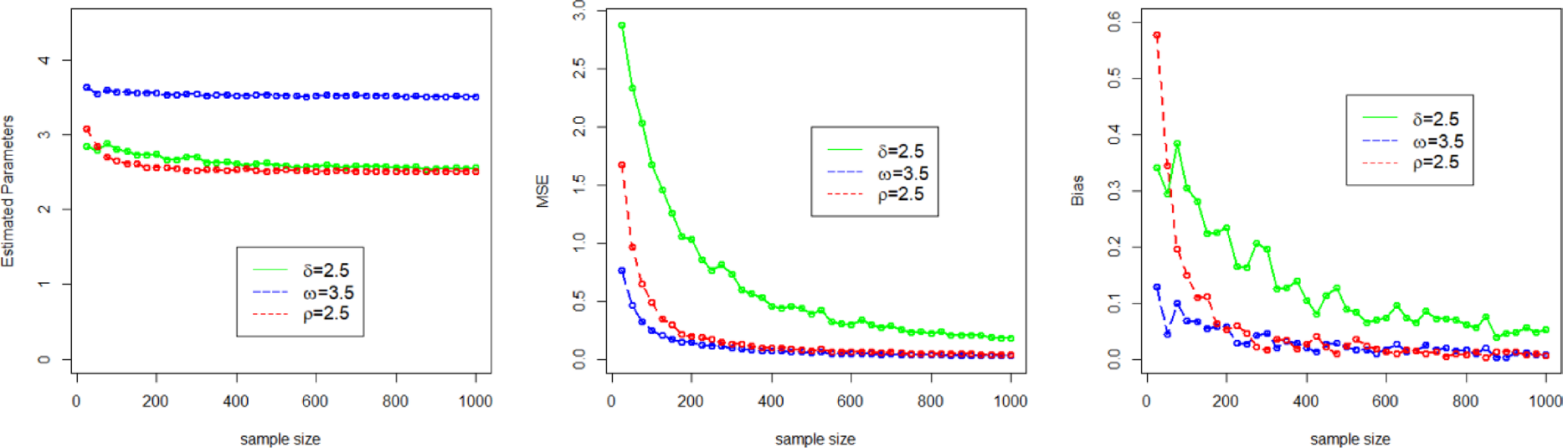
Graphical representation of the ${\Xi _{MLEs}}$, ${\Xi _{MSEs}}$, and ${\Xi _{Biases}}$ of the parameters $\delta$, $\omega$, and $\rho$ for Set I.

**Fig. 5:**
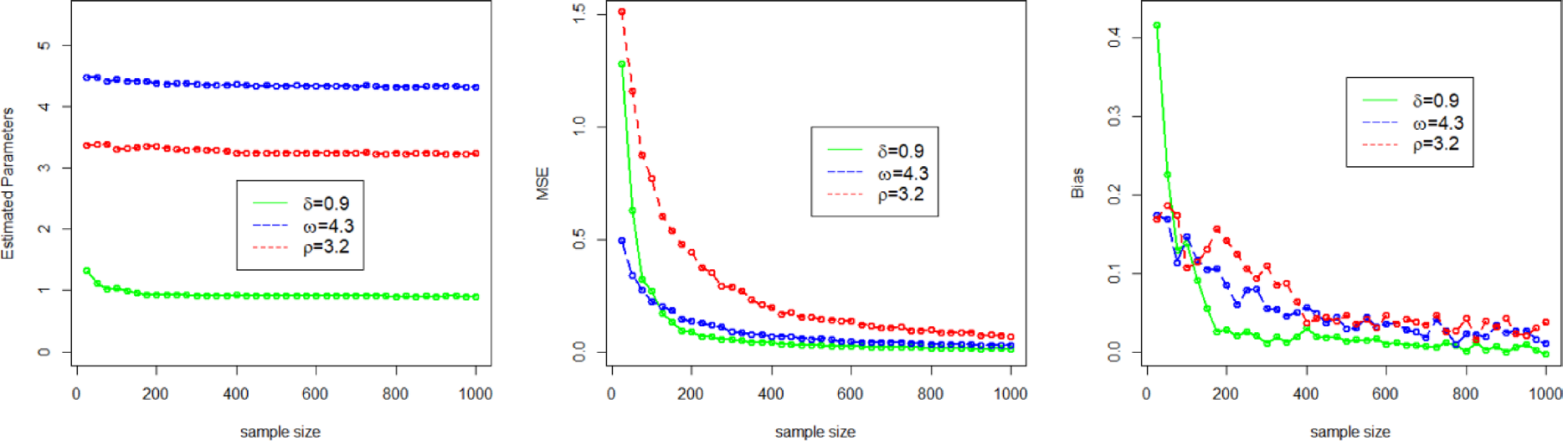
Graphical representation of the ${\Xi _{MLEs}}$, ${\Xi _{MSEs}}$, and ${\Xi _{Biases}}$ of the parameters $\delta$, $\omega$, and $\rho$for Set II.

**Fig. 6:**
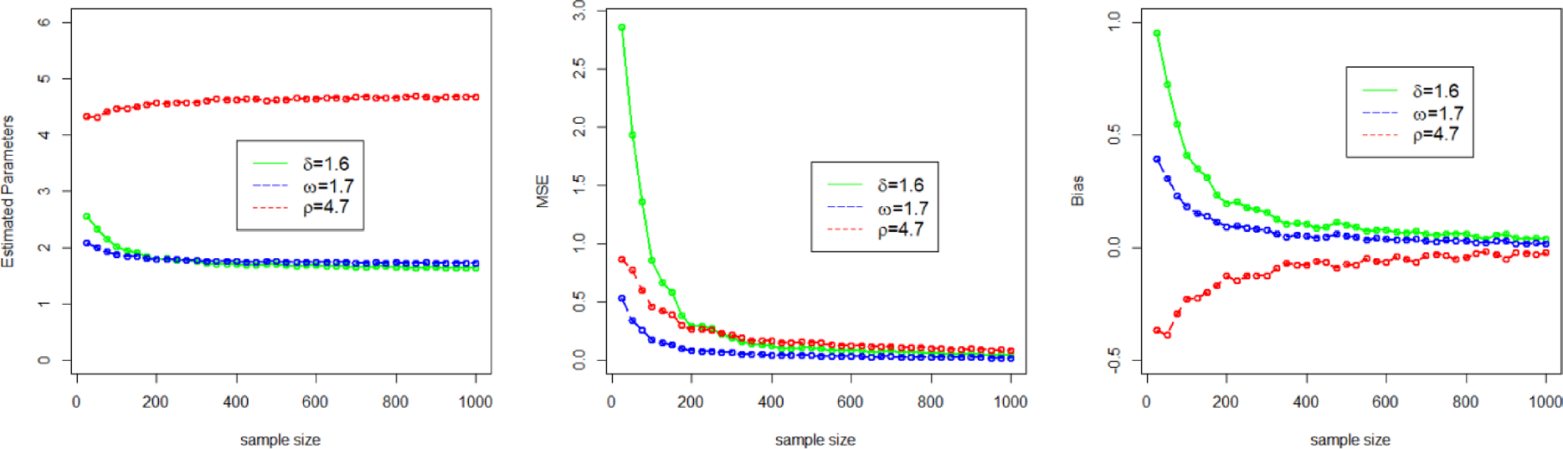
Graphical representation of the ${\Xi _{MLEs}}$, ${\Xi _{MSEs}}$, and ${\Xi _{Biases}}$ of the parameters $\delta$, $\omega$, and $\rho$for Set III.

**Fig. 7:**
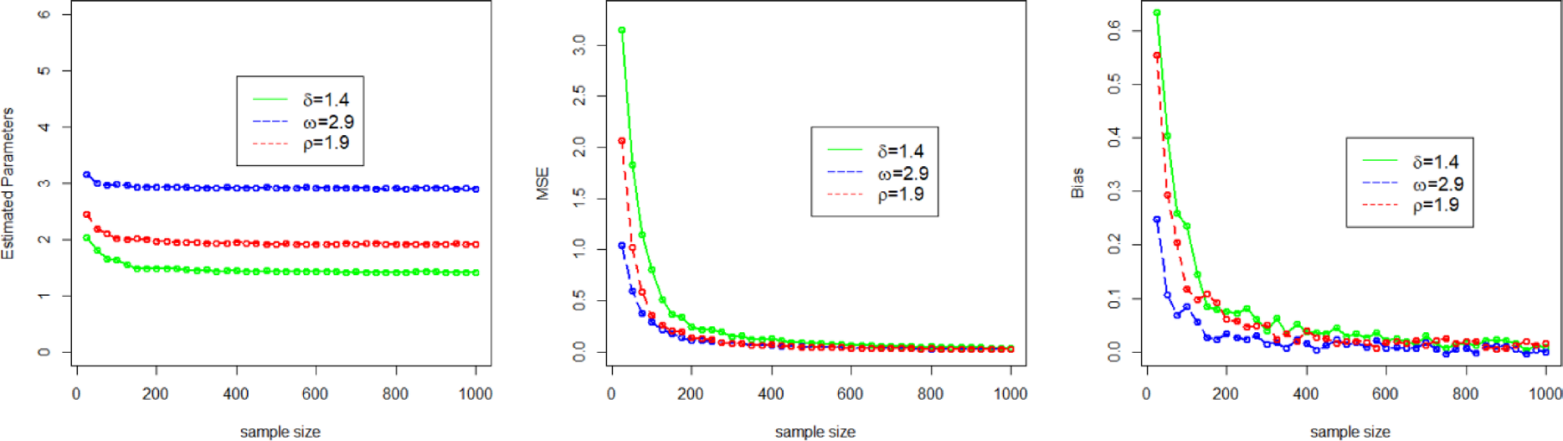
Graphical representation of the ${\Xi _{MLEs}}$, ${\Xi _{MSEs}}$, and ${\Xi _{Biases}}$ of the parameters $\delta$, $\omega$, and $\rho$for Set IV.

Similarly, for the convenience of understanding the simulation process graphically, we have also visualized the ${\Xi _{MLEs}}$, ${\Xi _{Biases}}$, and ${\Xi _{MSEs}}$ in Figs. 4, 5, 6 and 7. From the numerical and graphical illustration, we have observed the following result.

- As sample size n $\uparrow$, the ${\Xi _{MLEs}}$ for ${\delta _{MLE}}$, ${\omega _{MLE}}$, and ${\rho _{MLE}}$​ approach the true value.
- As sample size n $\uparrow$, the ${\Xi _{MSEs}}$ for ${\delta _{MLE}}$, ${\omega _{MLE}}$, and ${\rho _{MLE}}$decreases and approaches zero.
- As sample size n $\uparrow$, the ${\Xi _{Biases}}$ for ${\delta _{MLE}}$, ${\omega _{MLE}}$, and ${\rho _{MLE}}$decline.

These findings indicate that the MLE method is asymptotically efficient, consistent, and also satisfies the invariance property.

**Table 3:** ${\Xi _{MLEs}}$, ${\Xi _{MSEs}}$, and ${\Xi _{Biases}}$ for the estimated parameters for sets I and II.

n	Parameters	Set I($\delta$,$\omega$,$\rho$)=(2.5, 3.5, 2.5)			Set II($\delta$,$\omega$,$\rho$)=(0.9, 4.3, 3.2)		
		${\Xi _{MLEs}}$	${\Xi _{MSEs}}$	${\Xi _{Biases}}$	${\Xi _{MLEs}}$	${\Xi _{MSEs}}$	${\Xi _{Biases}}$
25	$\hat {\delta }$	2.842084	2.8778351	0.3420835	1.3163201	1.2820275	0.4163201
	$\hat {\omega }$	3.629724	0.7631049	0.1297244	4.473970	0.4955005	0.1739695
	$\hat {\rho }$	3.077381	1.6712779	0.5773807	3.368860	1.5149339	0.1688601
50	$\hat {\delta }$	2.794976	2.3351669	0.2949758	1.1262087	0.6276081	0.2262089
	$\hat {\omega }$	3.543819	0.4657410	0.0438192	4.469465	0.3390189	0.1694645
	$\hat {\rho }$	2.845080	0.9674905	0.3450798	3.386259	1.1585093	0.1862588
100	$\hat {\delta }$	2.805371	1.6783671	0.3053712	1.0383314	0.2709272	0.1383313
	$\hat {\omega }$	3.568163	0.2493127	0.0681625	4.446914	0.2231201	0.1469140
	$\hat {\rho }$	2.649642	0.4861586	0.1496415	3.306678	0.7727139	0.1066776
200	$\hat {\delta }$	2.735043	1.0355959	0.2350425	0.9276945	0.0890853	0.0276945
	$\hat {\omega }$	3.557753	0.1459824	0.0577534	4.384613	0.1362915	0.0846131
	$\hat {\rho }$	2.553774	0.2007239	0.0537737	3.342465	0.4421743	0.1424648
300	$\hat {\delta }$	2.695805	0.7330340	0.1958045	0.9101997	0.0540960	0.0101997
	$\hat {\omega }$	3.546894	0.1005486	0.0468938	4.355284	0.0911807	0.0552839
	$\hat {\rho }$	2.517254	0.1280364	0.0172536	3.309381	0.2887767	0.1093808
400	$\hat {\delta }$	2.605437	0.4593855	0.1054373	0.9300265	0.0411109	0.0300265
	$\hat {\omega }$	3.520864	0.0692183	0.0208636	4.356727	0.0695520	0.0567267
	$\hat {\rho }$	2.527465	0.0956741	0.0274649	3.237297	0.1950375	0.0372965
500	$\hat {\delta }$	2.588847	0.3929262	0.0888472	0.9130939	0.0288738	0.0130939
	$\hat {\omega }$	3.522285	0.0597890	0.0222847	4.329580	0.0530171	0.0295796
	$\hat {\rho }$	2.523971	0.0755366	0.0239711	3.246769	0.1541809	0.0467693
600	$\hat {\delta }$	2.572987	0.2936195	0.0729867	0.9098732	0.0246982	0.0098732
	$\hat {\omega }$	3.516974	0.0467301	0.0169742	4.335582	0.0466226	0.0355823
	$\hat {\rho }$	2.513391	0.0633184	0.0133907	3.246730	0.1353984	0.0467295
700	$\hat {\delta }$	2.585352	0.2904964	0.0853524	0.9072456	0.0194359	0.0072455
	$\hat {\omega }$	3.525375	0.0450217	0.0253753	4.317755	0.0397309	0.0177550
	$\hat {\rho }$	2.509205	0.0603742	0.0092054	3.234531	0.1074019	0.0345309
800	$\hat {\delta }$	2.562175	0.2184522	0.0621753	0.9002826	0.0170097	0.0002826
	$\hat {\omega }$	3.516531	0.0357700	0.0165312	4.322648	0.0352344	0.0226484
	$\hat {\rho }$	2.508671	0.0485433	0.0086709	3.243103	0.0974084	0.0431029
900	$\hat {\delta }$	2.545271	0.2050657	0.0452714	0.8990012	0.0148149	-0.0009988
	$\hat {\omega }$	3.503471	0.0315540	0.0034710	4.324958	0.0335770	0.0249584
	$\hat {\rho }$	2.512645	0.0453277	0.0126452	3.243055	0.0834364	0.0430546
1000	$\hat {\delta }$	2.553566	0.1839390	0.0535664	0.897482	0.0122737	-0.0025179
	$\hat {\omega }$	3.507570	0.0293473	0.0075698	4.311292	0.0268268	0.0112917
	$\hat {\rho }$	2.505565	0.0382036	0.0055646	3.238549	0.0685197	0.0385493

**Table 4:** ${\Xi _{MLEs}}$, ${\Xi _{MSEs}}$, and ${\Xi _{Biases}}$ for the estimated parameters for sets III and IV.

n	Parameters	Set III($\delta$,$\omega$,$\rho$)=(1.6, 1.7, 4.7)			Set IV($\delta$,$\omega$,$\rho$)=(1.4, 2.9, 1.9)		
		${\Xi _{MLEs}}$	${\Xi _{MSEs}}$	${\Xi _{Biases}}$	${\Xi _{MLEs}}$	${\Xi _{MSEs}}$	${\Xi _{Biases}}$
25	$\hat {\delta }$	2.553449	2.8554136	0.9534494	2.033755	3.15367642	0.6337547
	$\hat {\omega }$	2.091340	0.5279847	0.3913396	3.148167	1.03973427	0.4481671
	$\hat {\rho }$	4.332172	0.8621416	− 0.3678278	2.454411	2.06637179	0.5544112
50	$\hat {\delta }$	2.326336	1.9330286	0.7263360	1.803576	1.82882359	0.4035758
	$\hat {\omega }$	2.004071	0.3430793	0.3040707	3.006790	0.59300972	0.3467904
	$\hat {\rho }$	4.311083	0.7733345	− 0.3889172	2.193200	1.02379398	0.2931999
100	$\hat {\delta }$	2.008985	0.8567442	0.4089853	1.635143	0.80264728	0.2351433
	$\hat {\omega }$	1.880560	0.1751062	0.1805597	2.985045	0.29116902	0.2250449
	$\hat {\rho }$	4.469266	0.4576968	− 0.2307335	2.016769	0.35869136	0.1167691
200	$\hat {\delta }$	1.792276	0.2914938	0.1922761	1.476349	0.24405799	0.0763494
	$\hat {\omega }$	1.789705	0.0778298	0.0897048	2.934666	0.11498422	0.1346656
	$\hat {\rho }$	4.573276	0.2630955	− 0.1267235	1.961325	0.14271598	0.0613252
300	$\hat {\delta }$	1.753117	0.1868747	0.1531172	1.438973	0.15087956	0.0389733
	$\hat {\omega }$	1.774998	0.0600281	0.0749980	2.913585	0.07950586	0.0935851
	$\hat {\rho }$	4.572417	0.2175607	− 0.1275834	1.950534	0.08559808	0.0505343
400	$\hat {\delta }$	1.701362	0.1237998	0.1013622	1.437932	0.10784364	0.0299325
	$\hat {\omega }$	1.750500	0.0408962	0.0505004	2.915454	0.05761820	0.0754544
	$\hat {\rho }$	4.621099	0.1653605	− 0.0789011	1.938838	0.06126713	0.0388382
500	$\hat {\delta }$	1.697112	0.1087222	0.0971119	1.428124	0.07792538	0.0231239
	$\hat {\omega }$	1.749514	0.0364979	0.0495139	2.913295	0.04658763	0.0322948
	$\hat {\rho }$	4.623946	0.1459860	− 0.0760537	1.918959	0.04607907	0.0189587
600	$\hat {\delta }$	1.677748	0.0793898	0.0777479	1.420344	0.06030053	0.0153439
	$\hat {\omega }$	1.738471	0.0279023	0.0384713	2.907351	0.03700483	0.0173515
	$\hat {\rho }$	4.634496	0.1251585	− 0.0655044	1.917260	0.03733468	0.0132602
700	$\hat {\delta }$	1.657837	0.0770227	0.0578365	1.430507	0.05540334	0.0085073
	$\hat {\omega }$	1.727641	0.0276074	0.0276407	2.916803	0.03169859	0.0108030
	$\hat {\rho }$	4.665196	0.1157610	− 0.0348043	1.912907	0.03268996	0.0099069
800	$\hat {\delta }$	1.658430	0.0598639	0.0584299	1.415622	0.04587352	0.0050905
	$\hat {\omega }$	1.729779	0.0227647	0.0297793	2.906415	0.02725597	0.0078665
	$\hat {\rho }$	4.654062	0.1012150	− 0.0459383	1.918450	0.02854212	0.0064118
900	$\hat {\delta }$	1.660108	0.0543628	0.0601082	1.421858	0.03932110	0.0038582
	$\hat {\omega }$	1.728672	0.0216037	0.0286719	2.909922	0.02324580	0.0049224
	$\hat {\rho }$	4.646900	0.0985434	− 0.0531003	1.906041	0.02373819	0.0060409
1000	$\hat {\delta }$	1.636390	0.0416040	0.0363903	1.408946	0.02679972	0.0017847
	$\hat {\omega }$	1.717151	0.0174276	0.0171513	2.899337	0.01733669	0.0036521
	$\hat {\rho }$	4.676999	0.0810847	− 0.0230010	1.915708	0.01404223	0.0014101

## Applications

Here, we check the versatility and superiority of our newly developed distribution by analyzing three real data sets within the realm of the biomedical sector. We have implemented the proposed distribution on these datasets and then evaluated its performance (i.e., fitting power) against nine other existing probability distributions.

### Descriptions of the data sets

The first biomedical dataset comprises 44 observations, representing survival times of patients who have head and neck cancer diseases, obtained from Ünal et al.^32^. The observations of the first real dataset are: 12.20, 23.74, 23.56, 25.87, 37, 41.35, 31.98, 47.38, 55.46, 58.36, 63.47, 68.46, 78.26, 74.47, 81.43, 84, 92, 94, 110, 112, 119, 127, 130, 133, 140, 146, 155, 159, 173, 179, 194, 195, 209, 249, 281, 319, 339, 432, 469, 519, 633, 817, 725, 1776.

The second biomedical dataset comprises 36 observations, representing the death rate of COVID-19 patients in Canada over 36 days, from April 10 to May 15, 2020, and taken from Almetwally et al.^33^ and also available online, see the link [https://covid19.who.int/]. The observations of the second real dataset are: 3.1091, 3.3825, 3.1444, 3.2135, 2.4946, 3.5146, 4.9274, 3.3769, 6.8686, 3.0914, 4.9378, 3.1091, 3.2823, 3.8594, 4.0480, 4.1685, 3.6426, 3.2110, 2.8636, 3.2218, 2.9078, 3.6346, 2.7957, 4.2781, 4.2202, 1.5157, 2.6029, 3.3592, 2.8349, 3.1348, 2.5261, 1.5806, 2.7704, 2.1901, 2.4141, 1.9048.

Similarly, the third biomedical dataset comprises 20 observations, representing the lifetimes of twenty patients receiving analgesics, and the dataset is taken from Jamal et al.^34^, and also used by Shah et al.^35^. The observations of the third real dataset are: 1.1, 1.4, 1.3, 1.7, 1.9, 1.8, 1.6, 2.2, 1.7, 2.7, 4.1, 1.8, 1.5, 1.2, 1.4, 3.0, 1.7, 2.3, 1.6, 2.0.

**Fig. 8:**
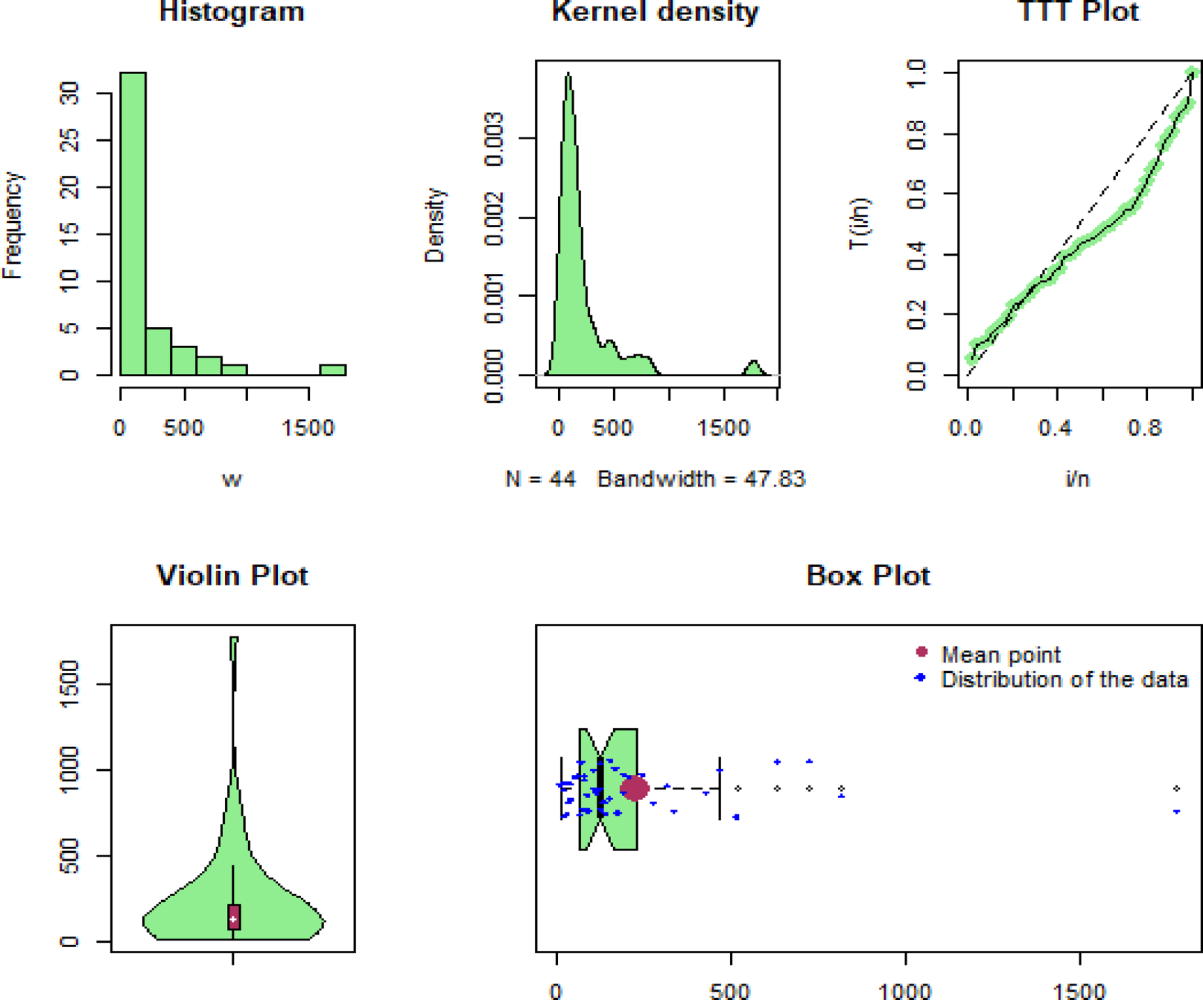
Graphical illustration of the first biomedical data set.

**Fig. 9:**
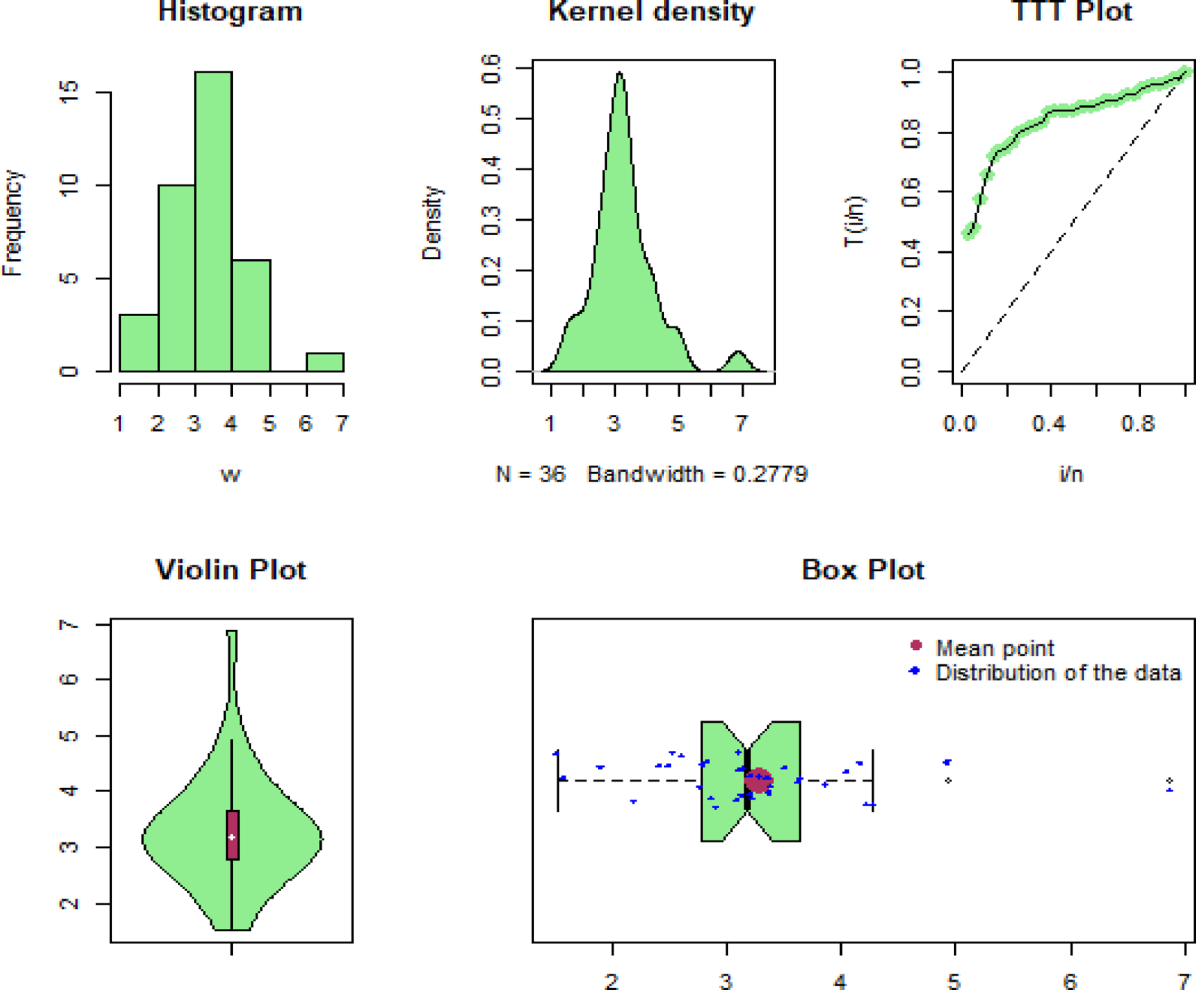
Graphical illustration of the second biomedical data set.

**Fig. 10:**
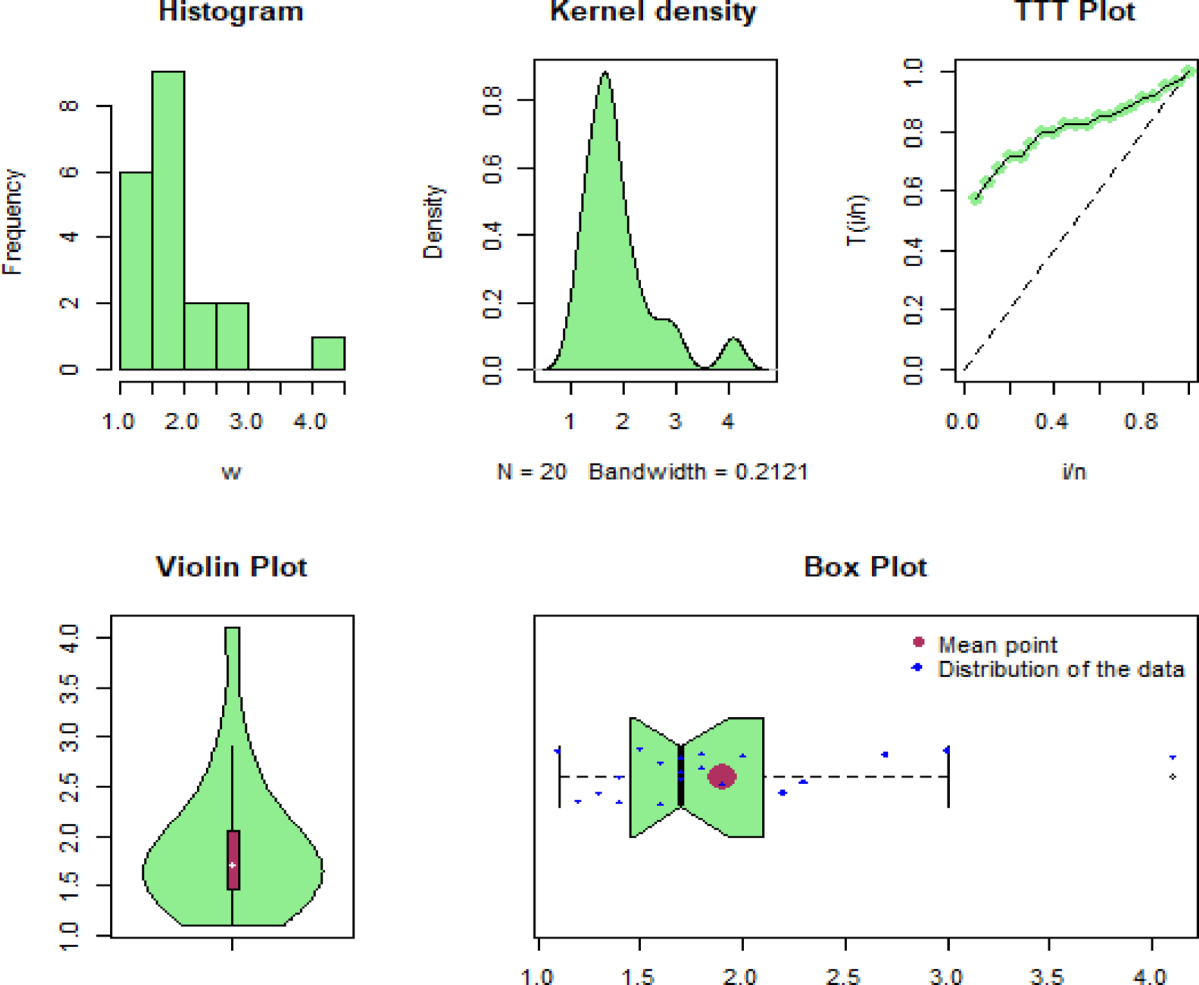
Graphical illustration of the third biomedical data set.

Some of the well-known descriptive measures of the first, second and third biomedical datasets are min=[ 12.20, 1.516, 1.100], max=[ 1776.00, 6.869, 4.100], 1st Qu=[ 67.21, 2.789, 1.475], 3rd Qu=[ 219.00, 3.637, 2.050], mean=[223.48, 3.282, 1.900], median=[ 128.50, 3.178, 1.700], variance=[ 93286.41, 0.9970656, 0.4957895], range=[ 1763.8, 5.3529, 3], skewness=[ 3.38382, 1.213916, 1.71975], and kurtosis=[ 16.5596, 6.151625, 5.924108]. Similarly, linked to these data sets, some graphical representations, including histograms, Kernel density, TTT-transformation, Violin, and Box plots, are visualized in Figs. 8, 9 and 10, respectively. From these Figures, we can see that the considered data sets for illustrative purposes are skewed to the right, and a skewed distribution can be highly effective for modeling these types of data sets.

### The competing distributions

To explore the utility and superiority of the NFOT-WD distribution over other probability distributions, we consider nine competing (or rival) distributions in the literature. The competing distributions include the following probability distributions, such as alpha power transformed Weibull (APT-W) of Dey et al.^6^, the traditional Weibull distribution proposed by Weibull^36^, the new exponential Weibull (NEx-W) of Shah et al.^37^, the new alpha power transformation Weibull (NAPT-W) of Albaatal et al.^38^, the Marshal Olkin Weibull (MO-W) distribution of Marshall and Olkin^4^, the novel flexible reduced logarithmic Weibull (FRL-W) model of Liu et al.^39^, the Sine alpha power Weibull (SAP-W) of Alghamdi et al.^40^, the alpha power Cosine Weibull (APC-W) of Alghamdi et al.^41^, and the Marshal Olkin Nadarajah Haghigh (MO-NH) of Muhammad et al.^42^. The CDFs of these competing distributions are.

- The APT-W distribution.
  $$F\left( {w;\alpha ,\phi } \right)=\frac{{{\alpha ^{\left( {1 - {e^{ - \omega {w^\rho }}}} \right)}} - 1}}{{\alpha - 1}};\quad \alpha ,\omega ,\rho \in {\Re ^+},w \in {\Re ^+}.$$
- The Weibull distribution.
  $$F\left( {w;\phi } \right)=1 - {e^{ - \omega {w^\rho }}};\quad \omega ,\rho \in {\Re ^+},w \in {\Re ^+}.$$
- The NEx-W distribution.
  $$F\left( {w;\phi } \right)=1 - \left( {\frac{{{e^{{e^{ - \omega {w^\rho }}}}} - 1}}{{e - {e^{ - \omega {w^\rho }}}}}} \right);\quad \omega ,\rho \in {\Re ^+},w \in {\Re ^+}.$$
- The NAPT-W distribution.
  $$F\left( {w;\alpha ,\phi } \right)=\frac{{\left( {1 - {e^{ - \omega {w^\rho }}}} \right){\alpha ^{\left( {1 - {e^{ - \omega {w^\rho }}}} \right)}}}}{\alpha };\quad \alpha ,\omega ,\rho \in {\Re ^+},w \in {\Re ^+}.$$
- The MO-W distribution.
  $$F\left( {w;\delta ,\phi } \right)=\frac{{\left( {1 - {e^{ - \omega {w^\rho }}}} \right)}}{{\delta +\left( {1 - \delta } \right)\left( {1 - {e^{ - \omega {w^\rho }}}} \right)}};\quad \delta ,\omega ,\rho \in {\Re ^+},w \in {\Re ^+}.$$
- The FRL-W distribution.
  $$F\left( {x;\delta ,\phi } \right)=1 - \frac{{\log \left\{ {\delta +1 - \delta \left( {1 - {e^{ - \omega {w^\rho }}}} \right)} \right\}}}{{\log \left( {1+\delta } \right)}};\quad \delta ,\omega ,\rho \in {\Re ^+},w \in {\Re ^+}.$$
- The SAP-W distribution.
  $$F\left( {x;\alpha ,\phi } \right)=\sin \left\{ {\frac{\pi }{2}\left( {\frac{{1 - {\alpha ^{\left( {1 - {e^{ - \omega {w^\rho }}}} \right)}}}}{{1 - \alpha }}} \right)} \right\};\quad \alpha ,\omega ,\rho \in {\Re ^+},w \in {\Re ^+}.$$
- The APC-W distribution.
  $$F\left( {w;\alpha ,\phi } \right)=\frac{{{\alpha ^{\cos \left( {\frac{\pi }{2}{e^{ - \omega {w^\rho }}}} \right)}} - 1}}{{\alpha - 1}};\quad \alpha ,\omega ,\rho \in {\Re ^+},w \in {\Re ^+}.$$
- The MO-NH distribution.
  $$F\left( {x;\delta ,\phi } \right)=\frac{{1 - {e^{\left\{ {1 - {{(1+\omega w)}^\rho }} \right\}}}}}{{1 - (1 - \delta ){e^{\left\{ {1 - {{(1+\omega w)}^\rho }} \right\}}}}};\quad \delta ,\omega ,\rho \in {\Re ^+},w \in {\Re ^+}.$$

### The evaluation criteria

Next, after selecting the competing models, we consider the discrimination measures (or model selection criteria) and goodness-of-fit outcomes along with their P-values. The discrimination measures (or information Criteria (IC)) are based on the Akaike Information Criteria (AIC, onward denoted by ${\Delta _1}$), Bayesian IC (BIC, onward denoted by ${\Delta _2}$), Consistent Akaike IC (CAIC, onward denoted by ${\Delta _3}$), Hannan-Quinn IC (HQIC, onward denoted by ${\Delta _4}$), while the goodness of fits outcomes are based on the Kolmogorov Smirnov (KS, onward denoted by ${\nabla _1}$), Anderson-Darling (AD, onward denoted by ${\nabla _2}$), Cramer von Mises (CM, onward dented by${\nabla _3}$), and their corresponding P-values. The mathematical formulas of these evaluation criteria are computed as.

- The AIC test statistics.

$${\Delta _1}=2p - 2\ell \left( \Theta \right);$$

- The BIC test statistics.

$${\Delta _2}=p\log (m) - 2\ell \left( \Theta \right);$$

- The CAIC test statistics.

$${\Delta _3}=\frac{{2mp}}{{m - p - 1}} - 2\ell \left( \Theta \right);$$

- The HQIC test statistics.

$${\Delta _4}=2p\log \left( {\log (m)} \right) - 2\ell \left( \Theta \right);$$

- The KS test statistics.

$${\nabla _1}={\sup _w}\left[ {{F_m}\left( w \right) - \hat {F}\left( w \right)} \right];$$

- The AD test statistics.

$${\nabla _2}= - m - \frac{1}{m}\sum\limits_{{i=1}}^{m} {\left( {2i - 1} \right)} \times \left[ {\log F\left( {{w_i}} \right)+\log \left( {1 - F\left( {{w_{i - m+1}}} \right)} \right)} \right];$$

- The CM test statistics.

$${\nabla _3}={\sum\limits_{{i=1}}^{m} {\left[ {F\left( {{w_i}} \right) - \frac{{2i - 1}}{{2m}}} \right]} ^2}+\frac{1}{{12m}}.$$

The values of the MLEs and the above evaluation measures are computed by using the package AdequacyModel in R-script with optim() and the BFGS algorithm. Generally, a distribution having the lowest values of${\Delta _1}$, ${\Delta _2}$, ${\Delta _3}$, ${\Delta _4}$, ${\nabla _1}$, ${\nabla _2}$, ${\nabla _3}$ and the highest P-value will be considered a good competitor for the considered data. Based on the above measures, it is exhibited that the NFOT-WD distribution has the lowest values of these evaluation measures and the highest P-value compared to the competing distributions.

### Analysis of the first dataset

**Fig. 11:**
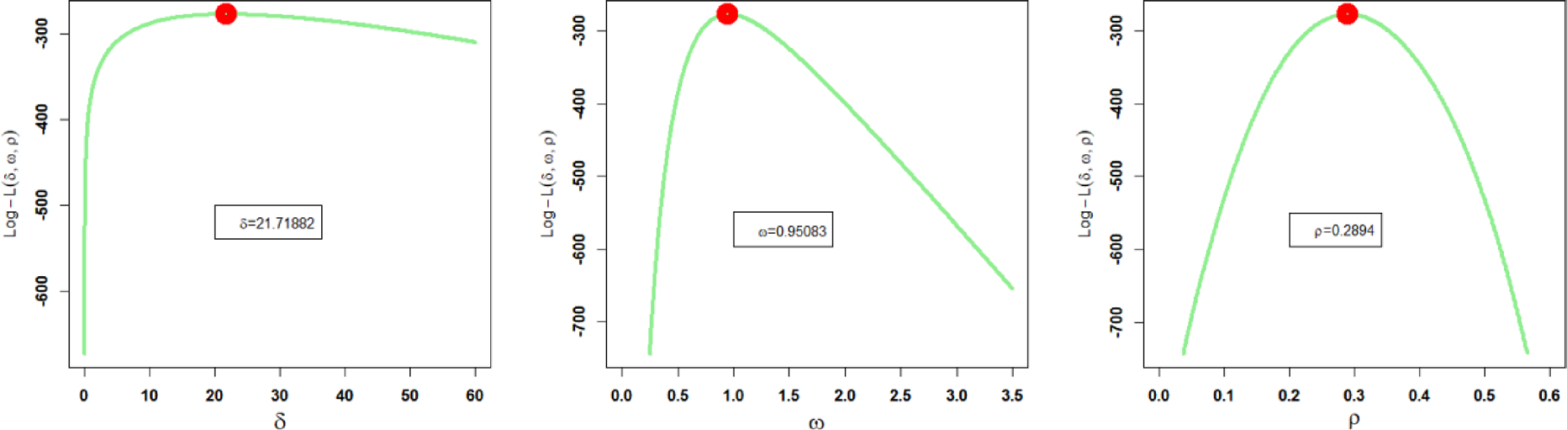
Profile plots of ${\hat {\delta }_{MLE}}$, ${\hat {\omega }_{MLE}}$ and ${\hat {\rho }_{MLE}}$of the NFOT-WD using the first dataset.

Corresponding to the first dataset, the estimated parameter values of the proposed and other competing probability distributions obtained through the method of MLEs, along with their related SEs (standard errors), are recorded in Table 5. Additionally, the profile plots of these estimated parameter values of the NFOT-WD are illustrated in Fig. 11. These profile likelihood plots demonstrate the uniqueness of all the estimated parameters and the maximization of the log likelihood function (LLF) of the NFOT-WD.

**Fig. 12:**
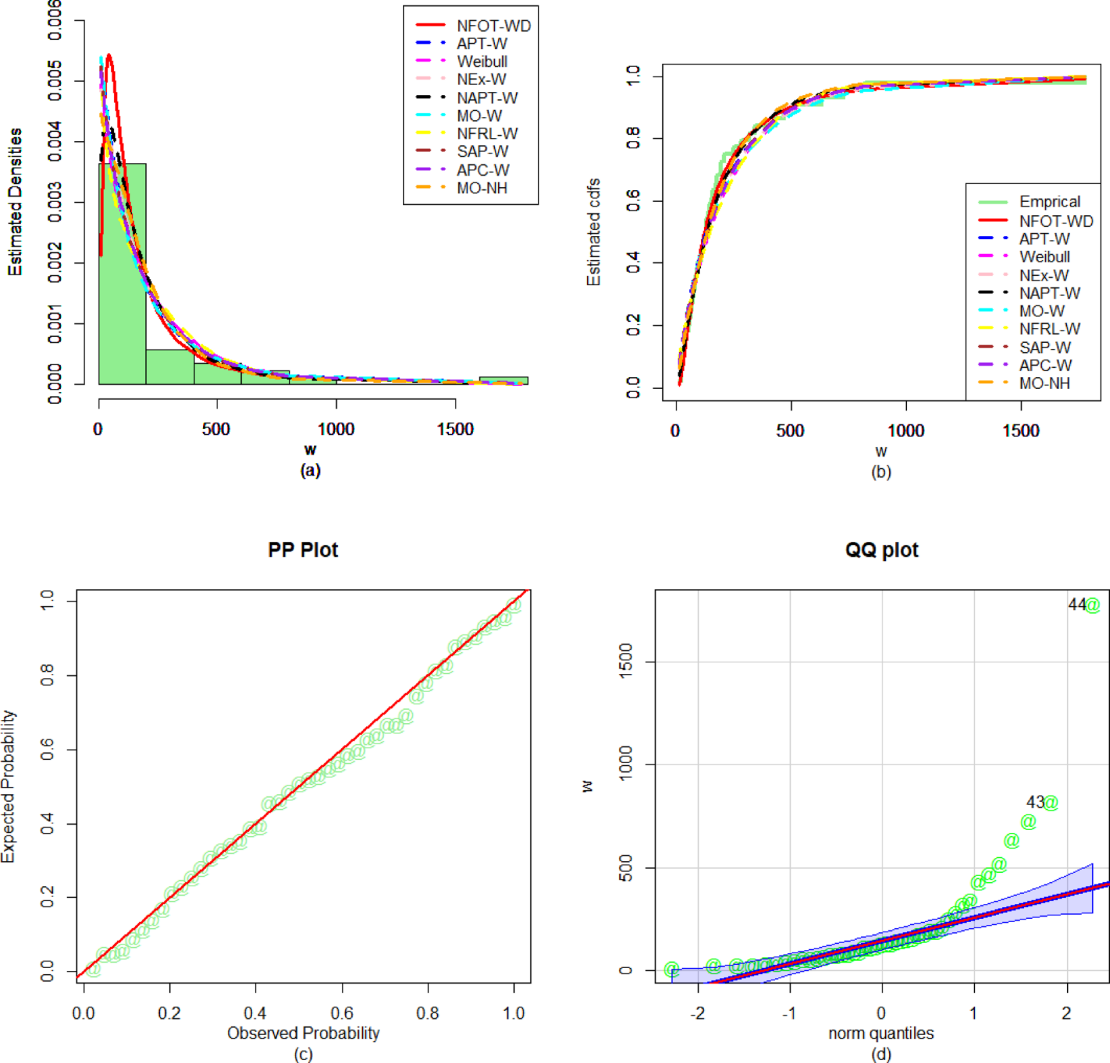
Visual illustrations of the (**a**) PDF fit, (**b**) CDF fit, (**c**) PP plot, and (**d**) QQ plot for the first biomedical dataset.

Similarly, to evaluate the performance of the fitted models for the first dataset, the numerical values of the discrimination measures (i.e., ${\Delta _1}$, ${\Delta _2}$, ${\Delta _3}$, and ${\Delta _4}$), goodness of fit outcomes (i.e., ${\nabla _1}$, ${\nabla _2}$, and${\nabla _3}$), and P-values are recorded in Table 6. From the numerical performance provided in Table 6, it is observed that the NFOT-WD is the best competitor distribution as it has the lowest values of all the evaluation measures and the highest P-value for the considered data set. Furthermore, to verify the versatility and superiority of the NFOT-WD, the graphical representations are illustrated in Fig. 12. For the graphical illustration, we consider the fitted PDF, fitted CDF, PP, and QQ plots of the NFOT-WD. From the graphical representations in Fig. 12, it is also demonstrated that the NFOT-WD provides a close fit to the considered dataset.

**Table 5:** MLEs with SE of the fitted distributions using the first data set.

Dist.	Par	SE	Par	SE	par	SE
NFOT-WD $\left( {\delta ,\omega ,\rho } \right)$	21.718823	21.7188234	0.9508359	0.9508359	0.2894048	0.2894048
APT-W$\left( {\alpha ,\omega ,\rho } \right)$	0.2450304	0.28574304	0.0032645	0.0011319	0.9927009	0.0597341
Weibull$\left( {\omega ,\rho } \right)$	0.0067712	0.00321090	0.9313115	0.0794288	–	–
NEx-W$\left( {\omega ,\rho } \right)$	0.0020126	0.00016523	1.0544436	0.0326874	–	–
NAPT-W$\left( {\alpha ,\omega ,\rho } \right)$	65.5118278	74.8036940	0.1622548	0.0735795	0.5157339	0.0721952
MO-W$\left( {\delta ,\omega ,\rho } \right)$	101.525793	111.049355	0.9653548	0.55610531	0.3213153	0.0726023
NFRL-W$\left( {\delta ,\omega ,\rho } \right)$	0.0731998	1.37453565	0.0089349	0.00858041	0.8864189	0.1197693
SAP-W$\left( {\alpha ,\omega ,\rho } \right)$	0.36507637	0.37588028	0.0030149	0.00082990	0.9162126	0.0583589
APC-W$\left( {\alpha ,\omega ,\rho } \right)$	0.40687490	0.42663455	0.0028092	0.00074947	0.9470984	0.0486874
MO-NH$\left( {\delta ,\omega ,\rho } \right)$	0.31550664	0.12471510	38.334407	84.3905609	0.9932587	3.3923852

**Table 6:** The numerical illustration of the fitted distributions for the first dataset.

Dist.	${\Delta _1}$	${\Delta _2}$	${\Delta _3}$	${\Delta _4}$	${\nabla _1}$	${\nabla _2}$	${\nabla _3}$	*P*-Values
NFOT-WD	560.7037	566.0563	561.3037	562.6887	0.058916	0.1221552	0.01899461	0.9957
APT-W	567.7712	573.1238	568.3712	569.7562	0.10551	0.5538716	0.09338685	0.6723
Weibull	567.6941	571.2625	567.9868	569.0175	0.12612	0.8142793	0.13983698	0.4494
NEx-W	564.8060	568.3743	565.0986	566.1293	0.10786	0.5356683	0.09006576	0.6459
NAPT-W	564.5400	569.8926	565.1400	566.5250	0.086975	0.3988119	0.06548925	0.8647
MO-W	567.5265	572.8791	568.1265	569.5115	0.093549	0.5958688	0.09757502	0.8019
NFRL-W	570.0054	575.3586	570.6054	571.9904	0.11962	0.7813038	0.13405291	0.5163
SAP-W	568.6198	573.9723	569.2198	570.6048	0.10572	0.6798777	0.11588338	0.6702
APC-W	568.8619	574.2144	569.4619	570.8469	0.10892	0.7171791	0.12232654	0.6344
MO-NH	566.7154	572.0684	567.3154	568.7004	0.088486	0.5623114	0.09299957	0.8511

### Analysis of the second dataset

**Fig. 13:**
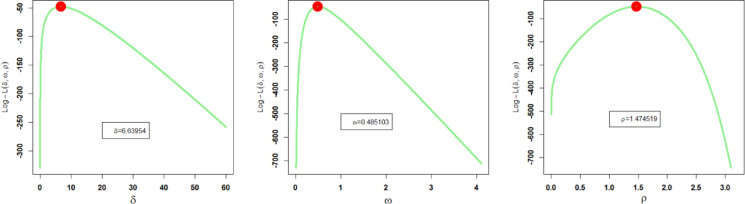
Profile plots of ${\hat {\delta }_{MLE}}$, ${\hat {\omega }_{MLE}}$ and ${\hat {\rho }_{MLE}}$of the NFOT-WD using the second data.

Similarly, corresponding to the second dataset, the estimated parameter values of the proposed and other competing probability distributions obtained through the method of MLEs, along with their related SEs (standard errors), are recorded in Table 7. Additionally, the profile plots of these estimated parameter values of the NFOT-WD are illustrated in Fig. 13. These profile likelihood plots demonstrate the uniqueness of all the estimated parameters and the maximization of the log likelihood function (LLF) of the NFOT-WD.

**Fig. 14:**
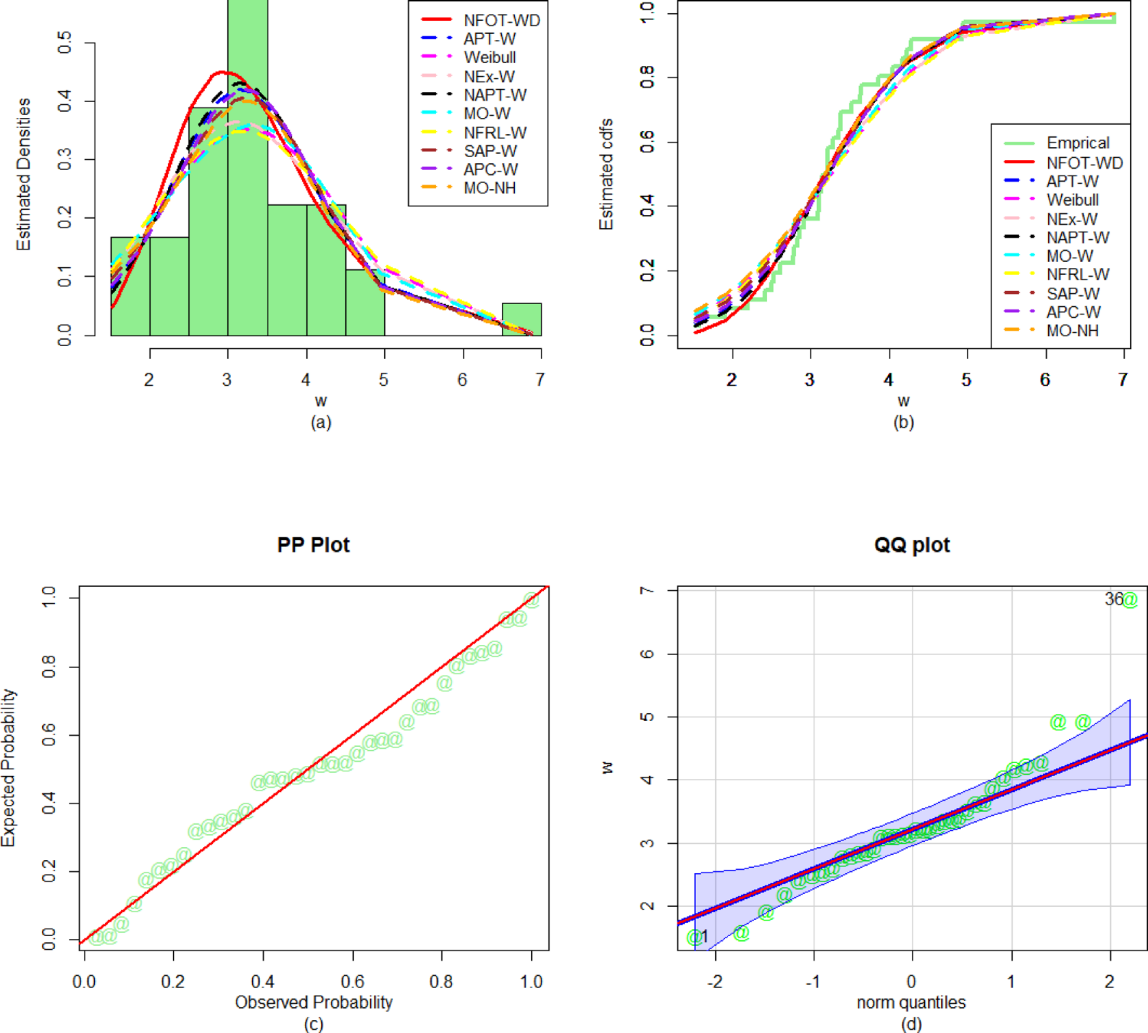
Visual illustrations of the (**a**) PDF fit, (**b**) CDF fit, (**c**) PP plot, and (**d**) QQ plot for the second biomedical dataset.

Likewise, to evaluate the performance of the fitted models for the second dataset, the numerical values of ${\Delta _1}$, ${\Delta _2}$, ${\Delta _3}$, ${\Delta _4}$,${\nabla _1}$, ${\nabla _2}$, and${\nabla _3}$, along with the P-values, are recorded in Table 8. From the numerical performance provided in Table 8, it is again observed that the NFOT-WD is the best competitor model as it has the lowest values of all the analytical measures and the highest P-value for the second considered data set. In addition to the numerical illustrations (Table 8), the versatility and superiority of the NFOT-WD are also graphically illustrated in Fig. 14. For the graphical illustration, we consider the plots of the fitted PDF, fitted CDF, PP, and QQ plots of the NFOT-WD distribution. From the graphical representations in Fig. 14, it is also confirmed that the NFOT-WD provides a better fit than the other competing distributions for the second dataset.

**Table 7:** MLEs with SE of the fitted distributions using the second data set.

Dist.	Par	SE	Par	SE	par	SE
NFOT-WD $\left( {\delta ,\omega ,\rho } \right)$	6.639536	6.5345088	0.485103	0.4797115	1.474519	0.5331800
APT-W$\left( {\alpha ,\omega ,\rho } \right)$	107.76803	126.706631	0.1846244	0.08744147	2.0084639	0.3055774
Weibull$\left( {\omega ,\rho } \right)$	0.0138762	0.00749783	3.3129913	0.35906864	–	–
NEx-W$\left( {\omega ,\rho } \right)$	0.0043943	0.00173718	3.7605715	0.28732874	–	–
NAPT-W$\left( {\alpha ,\omega ,\rho } \right)$	41.712422	53.3214711	0.1868754	0.09390862	2.0026549	0.3188230
MO-W$\left( {\delta ,\omega ,\rho } \right)$	0.5857972	0.56078013	0.0080901	0.01050733	3.4701938	0.5614739
NFRL-W$\left( {\delta ,\omega ,\rho } \right)$	0.5434849	0.51715873	0.0077136	0.00715463	3.5685026	0.4714242
SAP-W$\left( {\alpha ,\omega ,\rho } \right)$	105.689616	116.559600	0.1868405	0.09181126	1.7657507	0.2976094
APC-W$\left( {\alpha ,\omega ,\rho } \right)$	40.255376	44.7080178	0.0817562	0.0427068	2.0930279	0.3291227
MO-NH$\left( {\delta ,\omega ,\rho } \right)$	1.674270	0.4058982	45.562420	26.0618813	0.493748	0.2169030

**Table 8:** The numerical illustration of the fitted distributions for the second dataset.

Dist.	${\Delta _1}$	${\Delta _2}$	${\Delta _3}$	${\Delta _4}$	${\nabla _1}$	${\nabla _2}$	${\nabla _3}$	*P*-Values
NFOT-WD	102.0037	106.7543	102.7537	103.6618	0.10599	0.5352161	0.0918695	0.8134
APT-W	104.0491	108.7997	104.7991	105.7072	0.12367	0.6105432	0.1073291	0.6407
Weibull	106.9485	110.1156	107.3122	108.0539	0.14993	0.9916781	0.1728937	0.3933
NEx-W	104.3923	107.5594	104.7563	105.4977	0.13793	0.7625602	0.1320892	0.5000
NAPT-W	103.5698	108.3203	104.3198	105.2278	0.12395	0.5871649	0.1032307	0.6378
MO-W	108.3073	113.0579	109.0573	109.9654	0.13579	0.8456035	0.1465822	0.5203
NFRL-W	108.5318	113.2823	109.2818	110.1899	0.14789	0.9368068	0.1625074	0.4104
SAP-W	105.9926	110.7432	106.7426	107.6507	0.12334	0.7606668	0.1341807	0.6443
APC-W	104.8831	109.6336	105.6331	106.5412	0.12638	0.6781184	0.1191734	0.6133
MO-NH	106.2881	111.0387	107.0381	107.9462	0.12642	0.7114344	0.1260976	0.6128

### Analysis of the third dataset

**Fig. 15:**
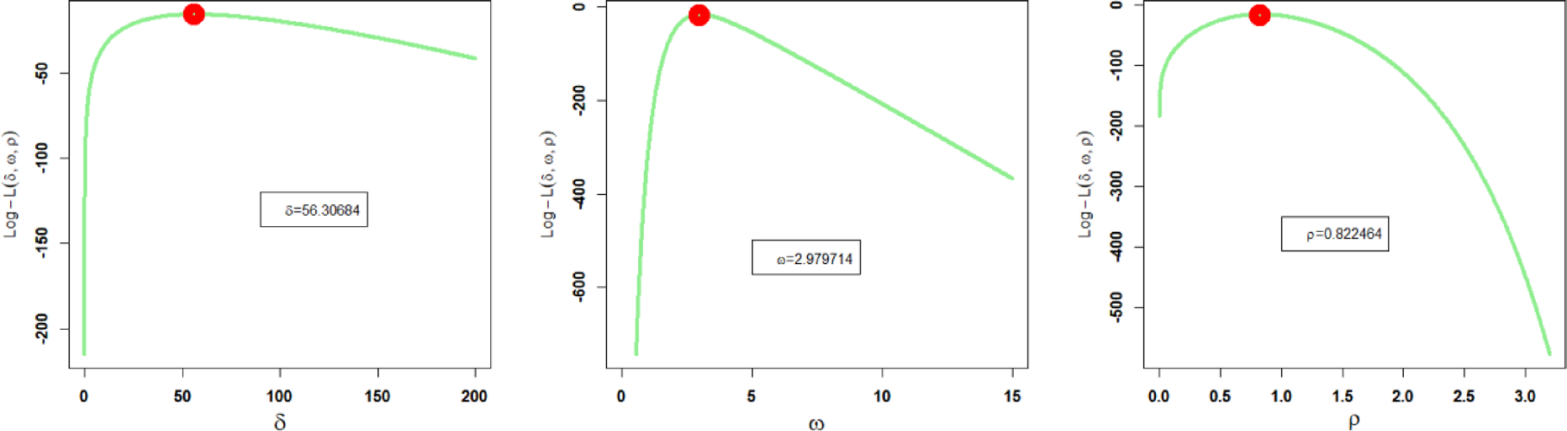
Profile plots of ${\hat {\delta }_{MLE}}$, ${\hat {\omega }_{MLE}}$ and ${\hat {\rho }_{MLE}}$of the NFOT-WD using the third dataset.

For the third dataset, the estimated parameter values of the proposed and other rival distributions obtained through the method of MLEs, along with their related SEs, are recorded in Table 9. Additionally, the profile plots of these estimated parameter values of the NFOT-WD are illustrated in Fig. 15. These profile likelihood plots demonstrate the uniqueness of all the estimated parameters and the maximization of the log likelihood function (LLF) of the NFOT-WD.

**Fig. 16:**
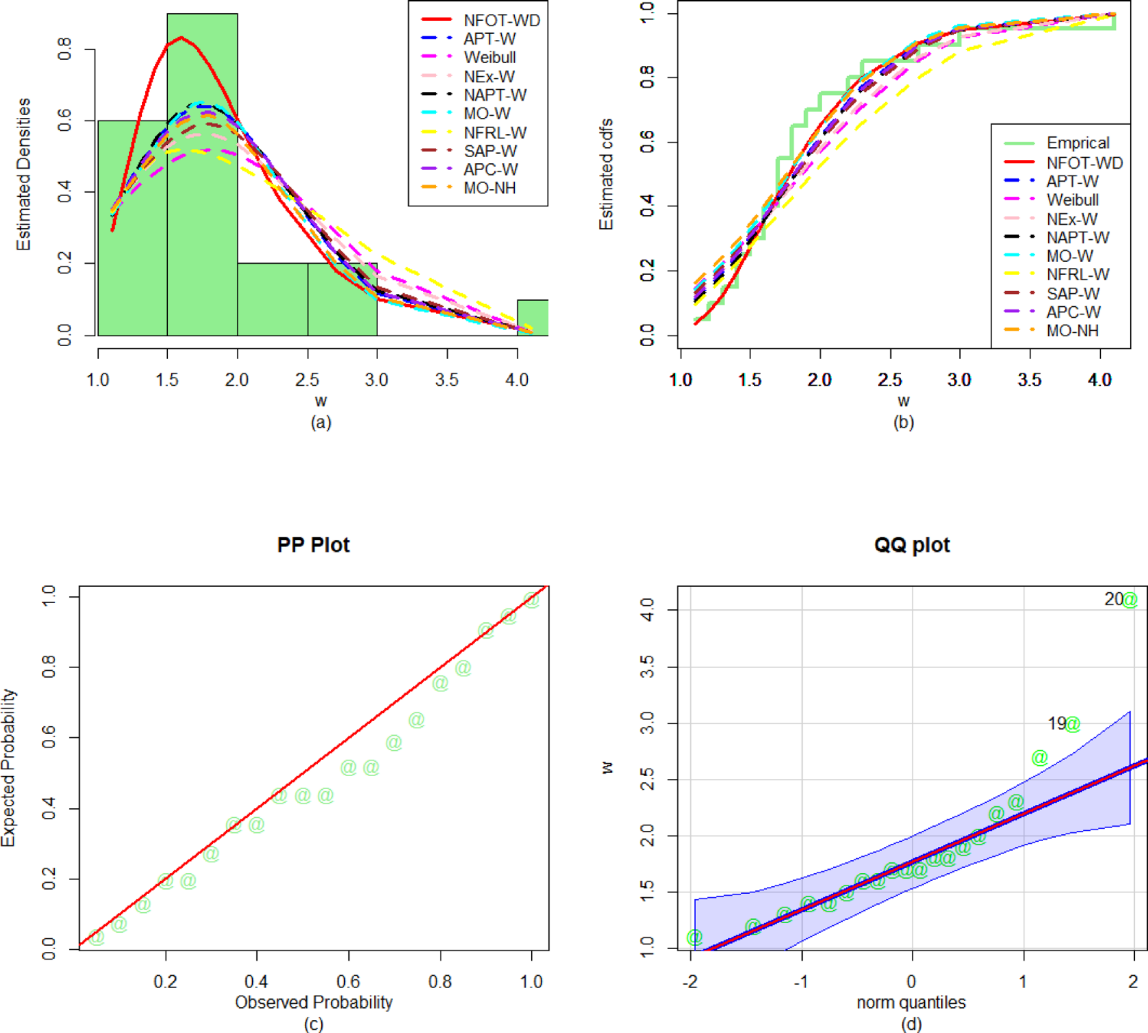
Visual illustrations of the (**a**) PDF fit, (**b**) CDF fit, (**c**) PP plot, and (**d**) QQ plot for the third biomedical dataset.

Similarly, to evaluate the performance of the fitted models for the third dataset, the numerical values of ${\Delta _1}$, ${\Delta _2}$, ${\Delta _3}$, ${\Delta _4}$,${\nabla _1}$, ${\nabla _2}$, and ${\nabla _3}$ along with the P-values are recorded in Table 10. Furthermore, to verify the versatility and superiority of the NFOT-WD, the graphical representations are also illustrated in Fig. 16. For the graphical illustration, we again consider the plots of the fitted PDF, fitted CDF, PP, and QQ plots of the NFOT-WD. From the numerical results, presented in Table 10, and the graphical representations in Fig. 16, it is demonstrated that the NFOT-WD provides a better fit (the proposed distribution closely follows the third dataset) than the other competing distributions.

**Table 9:** MLEs with SE of the fitted distributions using the third data set.

Dist.	Par	SE	Par	SE	Par	SE
NFOT-WD $\left( {\delta ,\omega ,\rho } \right)$	56.3068363	149.008928	2.9797144	2.4100065	0.8224639	0.4326042
APT-W$\left( {\alpha ,\omega ,\rho } \right)$	0.0158664	0.03368678	0.02073824	0.0150920	3.6200673	0.5587419
Weibull$\left( {\omega ,\rho } \right)$	0.1215765	0.05624065	2.78694570	0.4272109	–	–
NEx-W$\left( {\omega ,\rho } \right)$	0.0460279	0.02293551	3.32337639	0.4791495	–	–
NAPT-W$\left( {\alpha ,\omega ,\rho } \right)$	53.6526320	77.2953052	0.73458920	0.2384752	1.6611311	0.2985984
MO-W$\left( {\delta ,\omega ,\rho } \right)$	90.104621	114.773184	2.52647900	1.0702830	1.0127960	0.2704508
NFRL-W$\left( {\delta ,\omega ,\rho } \right)$	0.9862393	0.01743541	0.00448578	0.0042957	4.8278000	0.6944573
SAP-W$\left( {\alpha ,\omega ,\rho } \right)$	0.0537121	0.12159160	0.02150370	0.0173328	3.0978320	0.4999307
APC-W$\left( {\alpha ,\omega ,\rho } \right)$	0.0155368	0.03369849	0.01400824	0.0100632	3.4545281	0.5367068
MO-NH$\left( {\delta ,\omega ,\rho } \right)$	1.0720617	0.38220080	58.5837410	55.685517	2.022742	1.7582338

**Table 10:** The numerical illustration of the fitted distributions for the third dataset.

Dist.	${\Delta _1}$	${\Delta _2}$	${\Delta _3}$	${\Delta _4}$	${\nabla _1}$	${\nabla _2}$	${\nabla _3}$	*P*-values
NFOT-WD	37.89799	40.88519	39.39799	38.48112	0.13142	0.2663213	0.0457203	0.8801
APT-W	43.41718	46.40438	44.91718	44.00031	0.15791	0.7784439	0.1324222	0.7009
Weibull	45.17281	47.16427	45.87869	45.56156	0.18495	1.0928755	0.1857121	0.5007
NEx-W	42.93560	44.92706	43.64148	43.32435	0.18129	0.9042441	0.1521853	0.5267
NAPT-W	43.20095	46.18815	44.70095	43.78409	0.16321	0.7472262	0.1262447	0.6611
MO-W	44.65314	47.64034	46.15314	45.23627	0.14402	0.8174189	0.1392677	0.8012
NFRL-W	44.70328	47.69047	46.20328	45.28641	0.22294	0.8522219	0.1402434	0.2732
SAP-W	45.21186	48.19905	46.71186	45.79499	0.16519	0.9243609	0.1577305	0.6462
APC-W	43.94548	46.93268	45.44548	44.52861	0.15957	0.8242832	0.1405009	0.6885
MO-NH	45.28827	48.27547	46.78827	45.87141	0.16167	0.8456694	0.1441994	0.6732

## Conclusion

In this article, we have introduced a novel approach for deriving new heavy-tailed distributions, which we have aptly named the NFOT-G family of distributions. The NFOT-GFD method has been applied to the Weibull distribution, and a new distribution known as the NFOT-WD is introduced. The PDF and HF plots with diverse parameter initial values are illustrated graphically. The PDF plots of the NFOT-WD can exhibit positively skewed behavior, which is valuable for modeling heavy-tailed data. Similarly, the HF of the NFOT-WD can also take different forms, such as increasing, decreasing, unimodal, J-shaped, reverse J-shaped, S-shaped, and bathtub-shaped. For the NFOT-WD, various distributional (or structural) properties such as moments, moment generating function, characteristic function, mean deviation from median, and order statistics are explored. To examine the tail behavior of the proposed distribution, we have utilized the actuarial measures, including Value at Risk and Tail Value at Risk. These actuarial measures reveal that the proposed distribution can effectively model heavy-tailed datasets. To estimate the unknown parameters of the proposed distribution, the maximum likelihood method is employed. To test the effectiveness of the applied estimation method for the proposed distribution, a comprehensive simulation study is conducted, and it is observed that as the sample size increases, the MSEs and Biases decrease and approach zero. Lastly, three biomedical data sets are considered to check the practical performance of the proposed distribution. The flexibility of the proposed distribution is compared to other existing distributions (i.e., APT-W, Weibull, NEx-W, NAPT-W, MO-W, and NFRL-W) using various model selection criteria and goodness-of-fit metrics. Based on numerical and graphical illustrations, it is concluded that the NFOT-WD is the prominent alternate model for the considered biomedical datasets. We expect that this new method will encourage scholars to apply their findings in biomedical sciences as well as in various other fields of study.

## Data Availability

The data supporting this study’s findings are available within the article.
